# Longitudinal monitoring of circulating tumour DNA improves prognostication and relapse detection in gastroesophageal adenocarcinoma

**DOI:** 10.1038/s41416-020-1002-8

**Published:** 2020-07-28

**Authors:** Mark R. Openshaw, Ali Abdulnabi Suwaidan, Barbara Ottolini, Daniel Fernandez-Garcia, Cathy J. Richards, Karen Page, David S. Guttery, Anne L. Thomas, Jacqui A. Shaw

**Affiliations:** Leicester Cancer Research Centre, University of Leicester, Leicester, UK

**Keywords:** Gastric cancer, Prognostic markers, Cancer genomics, Tumour biomarkers, Cancer genetics

## Abstract

**Background:**

Gastroesophageal adenocarcinoma (GOA) has poor clinical outcomes and lacks reliable blood markers. Here we present circulating tumour DNA (ctDNA) as an emerging biomarker.

**Methods:**

Forty patients (17 palliative and 23 curative) were followed by serial plasma monitoring. Primary tumour DNA was analysed by targeted next-generation sequencing to identify somatic single-nucleotide variants (SNVs), and Nanostring nCounter^®^ to detect copy number alterations (CNAs). Patient-specific SNVs and CNA amplifications (CNA^amp^) were analysed in plasma using digital droplet PCR and quantitative PCR, respectively.

**Results:**

Thirty-five patients (13 palliative, 22 curative) had ≥1 SNVs and/or CNA^amp^ detected in primary tumour DNA suitable for tracking in plasma. Eighteen of 35 patients (nine palliative, nine curative) had ≥1 ctDNA-positive plasma sample. Detection of postoperative ctDNA predicted short RFS (190 vs 934 days, HR = 3.7, *p* = 0.028) and subsequent relapse (PPV for relapse 0.83). High ctDNA levels (>60.5 copies/ml) at diagnosis of metastatic disease predicted poor OS (90 vs 372 days, HR = 11.7 *p* < 0.001).

**Conclusion:**

Sensitive ctDNA detection allows disease monitoring and prediction of short OS in metastatic patients. Presence of ctDNA postoperatively predicts relapse and defines a ‘molecular relapse’ before overt clinical disease. This lead time defines a potential therapeutic window for additional anticancer therapy.

## Background

Worldwide there were over 1.6 million cases of stomach and oesophageal cancer in 2018 with 1.2 million deaths.^1^ Gastroesophageal adenocarcinoma (GOA), which includes oesophageal adenocarcinoma, gastroesophageal junctional adenocarcinoma and stomach cancer, comprises an increasing proportion of these cancers, particularly in Western Europe.^2,3^ Recent genetic and molecular research has demonstrated that GOA is a single disease entity with molecular subgroups.^4^ While advances in the treatment of GOA, including multi-agent chemotherapy and trastuzumab, have improved survival in metastatic disease, median overall survival (OS) remains less than 12 months^5,6^ and 5-year survival rates for locally advanced disease are 24–38%.^7–10^ Therefore, GOA has significant unmet clinical need, which requires greater understanding of the disease process, and improvement in disease monitoring, to help improve survival.

Circulating tumour DNA (ctDNA), the tumour-derived fraction of plasma cell free DNA (cfDNA), has been widely studied as a method for monitoring cancers^11–14^ and early detection of recurrence.^15,16^ ctDNA presence is demonstrated by the detection of tumour-specific variants in plasma cfDNA. However, ctDNA analysis may detect variants not present in single GOA tumour biopsies,^17^ that are present when multiple tumour biopsies are analysed.^18^ This demonstrates the inherent ability of ctDNA to reflect underlying tumour heterogeneity, which has been reported in several cancers,^19^ and indicates ctDNA may become an important tool in disease monitoring.

Genetic analysis of GOA has revealed significant genetic heterogeneity, including high levels of somatic alterations comprising single-nucleotide variants (SNVs), short INDELS, translocations and copy number alterations (CNAs).^4,20,21^ Previous studies have demonstrated detection of a subset of these variants in plasma cfDNA, using next-generation sequencing (NGS), droplet digital (dd)PCR and quantitative (q)PCR.^18,22–25^ However, these studies did not fully evaluate the importance of ctDNA detection in postoperative samples and did not monitor ctDNA levels during therapy. More recently Maron et al.^26^ used the Guardant360 test to detect ctDNA in a large cohort of 1630 patients and showed that ctDNA analysis could interrogate the genomic landscape of GOA tumours. In a smaller clinically annotated cohort of 22 patients Maron et al.^26^ showed that presence of postsurgical ctDNA was associated with reduced disease-free survival and that patients that had reduction in ctDNA levels following first-line therapy had improved survival. In support of these findings a number of studies have reported that detection of ctDNA may allow disease tracking in both the metastatic and adjuvant setting in specific molecular or clinical subgroups.^24–27^ Given these findings there is increasing evidence that ctDNA may have clinical application in patients with GOA that have curable or metastatic disease.

Here, we profiled tumour-specific SNVs and CNA amplifications (CNA^amp^) in 116 serial plasma samples from 40 patients with GOA using high-sensitivity detection methods (targeted NGS, ddPCR and qPCR) applicable to most diagnostic service laboratories. We demonstrate subsequent detection and longitudinal monitoring of ctDNA, including detection of molecular relapse and prediction of relapse-free survival (RFS) following surgery, and prediction of OS in metastatic patients.

## Methods

### Patient and samples

Forty patients with GOA were recruited between November 2015 and November 2017, with follow-up until March 2019. Patients were recruited in accordance with the Declaration of Helsinki and consented to sample storage at the University of Leicester Cancer Research Biobank, UHL11274 (REC: 13/EM/0196). Primary tumour (FPPE or Fresh Frozen) and up to seven serial plasma samples were collected. Plasma samples were prepared from up to 25 ml of venous blood, collected into EDTA tubes. Samples were stored on ice and processed within 2 h. Plasma was prepared from blood with an initial centrifugation at 1000 × *g* at 4 °C for 10 min, followed by plasma separation and a further centrifugation at 2000 × *g*.^28^ Plasma samples were stored at −80 °C until extraction. The buffy coat (lymphocyte) layer was also separated during blood processing and stored at −80 °C. FFPE DNA (31 samples) was extracted using the GeneRead™ DNA FFPE kit, and Fresh frozen DNA (nine samples) using the QIAamp^®^ DNA mini-kit as per manufacturer’s instructions. DNA was extracted from 200 µl of buffy coat using the QIAamp^®^ Blood mini-kit. Tumour and buffy coat DNA were quantified using the Quibit^®^ broad range kit. Plasma cfDNA was extracted from 3 ml of plasma using the QIAamp^®^ Circulating Nucleic Acid Kit and quantified using *GAPDH* qPCR as described previously.^29^

### Next-generation sequencing

A six-gene Ampliseq panel was designed for tumour next-generation sequencing (NGS). The most commonly mutated genes in GOA were identified using publicly available whole-exome sequencing GOA data^20,21,30^ (Supplementary Fig. 1). On review of the data in cBioportal, those genes with recurrent mutation hotspots^31,32^ were evaluated for inclusion in the panel. Inclusion of a gene in the designed panel required either (i) high frequency (>10%) of mutation in one GOA subtype, and presence across all subtypes of GOA, or (ii) frequency of mutation >5% in one GOA subtype and ability to include mutation hotspots in ≤3 amplicons. Amplicons were designed using Ion Ampliseq Designer (v.5.0)^33^ to target regions containing identified hotspots. The panel included 139 hotspots in *TP53, PIK3CA, ARID1A, SMAD4, RHOA* and *KRAS* (Supplementary Fig. 2). Twenty nanogram of tumour/plasma cfDNA was sequenced using the designed panel on the IonTorrent PGM™ as described previously.^34^ Sequencing data were analysed using IonTorrent Suite™ software (v.5.6) and IGV (v.2.3.82). Variant calling required a quality score >20, reference reads >30, mutant reads >5, variant allele frequency (VAF) > 2%, strand bias <0.45 and location >10 bases from the amplicon end, unless also confirmed by ddPCR.

### ddPCR

Commercial BioRad ddPCR assays were used according to manufacturer’s guidelines (Supplementary Fig. 3). Where unavailable, ddPCR assays were designed with Primer3 (http://primer3.ut.ee) using a 90–120 bp region flanking the base(s) of interest and reviewed with Oligoanalyser (https://www.idtdna.com). Designed primers were checked for efficiency and generation of a single product.^35^ Designed primer/probe assays (Supplementary Fig. 4a) were optimised using 5 ng of tumour DNA (positive for the variant of interest) over a 10 °C temperature gradient around the predicted annealing temperature. 5–20 ng of template DNA was used per ddPCR reaction and analysed with Quantasoft (v.1.3.2). Each reaction involving plasma cfDNA used patient tumour DNA as a positive control, and patient germline DNA (buffy coat) and human genomic DNA as negative controls in addition to a nontemplated control. Detection of three positive mutant droplets was required to call a positive ctDNA sample. Replicates were undertaken for borderline results (positive mutant droplets 1–6) using 20 ng of plasma cfDNA where sufficient plasma cfDNA was available.

### NanoString nCounter^®^ copy number analysis

The NanoString nCounter^®^ v2 platform was used for tumour DNA CNA analysis^36^ using 400 ng of tumour and normal tissue DNA. Results were normalised using the nSolver™ software as per company instructions. Probe counts in tumour were compared to paired normal tissue counts to generate copy number. Where paired tissue was unavailable (*n* = 12) average counts from eight normal tissue samples was used for FFPE samples, and the Coriell Institute DNA sample NA10854 for fresh frozen tissue (genotype data: http://www.internationalgenome.org/data-portal/sample/NA10854).

### Real-time quantitative PCR

Real-time qPCR was used to analyse gene amplification of *ERBB2, MYC, CCND1, CCNE1* and *VEGFA* in 0.5–3 ng plasma cfDNA as described previously^37^ using two reference genes: *GAPDH* and *CNTNAP1*. In short, detection of gene amplification used relative quantification (RQ) real-time qPCR, by calculating the difference in cycle threshold (Ct) value for a reference (nonamplified) gene (Ct:R) against the Ct value of the gene of interest (Ct:GI), using the equation: copy number (CN) = (2^(Ct:GI – Ct:R)^) × 2. As primers for the amplicon in the gene of interest and the reference gene will not have identical efficiency, an internal calibration step was included. Reactions were run simultaneously against the experimental DNA (ExDNA) sample and buffy coat DNA (BCDNA) from the same patient, allowing a difference in Ct to be calculated for each sample. Therefore if ‘Ct:GI – Ct:R’ is termed dCt, CN = (2^(dCt:ExDNA – dCt:BCDNA)^) × 2 using the internal calibration step. All experiments were carried out in triplicate and the average Ct value used to calculate CN. Supplementary Fig. 5 contains detailed reaction conditions. The *ERBB2* assay was as described previously^35^ and the other gene assays were designed and validated as for SNV assays (Supplementary Fig. 4b). Based on a previous study^38^ a CN threshold of ≥3 was applied for detection of gene amplification in plasma cfDNA.

### Statistical analysis

All data are presented descriptively as means, medians or proportions. Correlations between targeted sequencing VAF and ddPCR VAF results, and between NanoString nCounter^®^ CNA and qPCR CNA results were tested using a linear regression model with a coefficient of determination (R^2^) to determine the Goodness of Fit.

Kaplan–Meier estimator and Cox regression models were used to assess RFS and OS. Each model was constructed using the counting process notation (start, end, event).^39^ Date of surgery was taken as the start date for curative patient RFS analysis, and date of diagnosis of metastatic disease as start date for metastatic/palliative patient OS analysis. Date of last follow-up, date of progression or date of death was considered the end, with censoring date of 31st March 2019. Cox proportional-hazards regression analysis was used to estimate hazard ratios for RFS and OS. An optimal ctDNA concentration cut-off point for OS less than 6 months in metastatic patients was determined using Receiver Operating Curve (ROC) analysis.

All *p*-values were two-sided and a *p*-value < 0.05 was considered statistically significant. Statistical analyses were performed using GraphPad Prism (version 7) apart from the survival analyses, which were performed using SPSS (version 25). SNV and CNA data were summarised using OncoPrinter (Version 3.2.11).^31^

## Results

### Patient characteristics

**Fig. 1:**
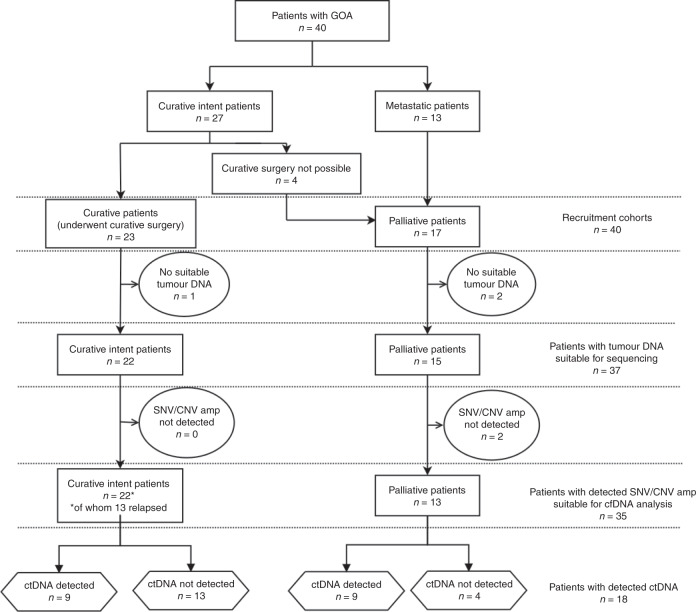
Consort diagram of patient workflow. Showing recruitment cohorts and summarising tumour DNA/total cfDNA analysis and ctDNA detection.

A cohort of 40 patients was recruited including 17 palliative patients and 23 undergoing curative treatment. Four patients initially treated with neoadjuvant chemotherapy were unable to undergo curative surgery and were included in the palliative cohort (Fig. 1). The median age was 68 years (range 47–90) (Demographic data: Supplementary Fig. 6).

### Detection of high-frequency mutations in tumour DNA

**Fig. 2:**
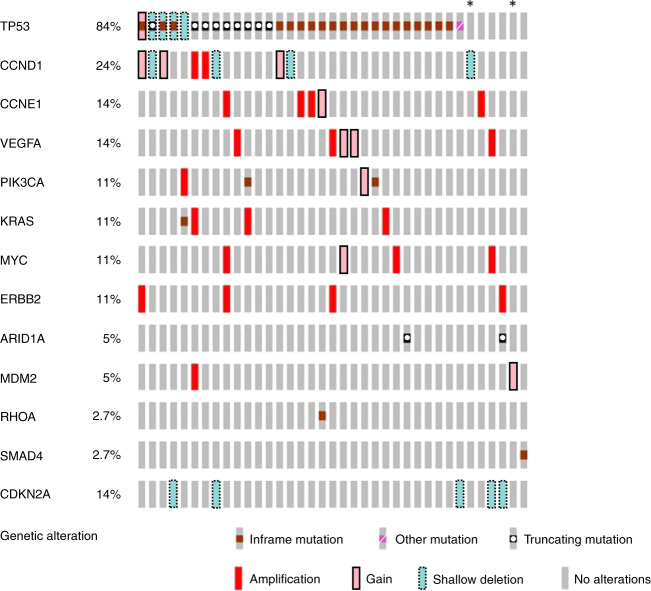
Oncoprint of gene mutations and copy number changes in 37 GOA tumours. Results for the genes included in the six-gene SNV panel and genes most frequently showing a variation in copy number within this cohort. Results are the combined findings from tumour DNA NGS SNV analysis and Nanostring CNA analysis. *Patient with no detected SNV or CNA amplification. Amplification = CN ≥ 4, Gain = CN ≥ 3, shallow deletion = CN < 1.5.

All 40 tumour DNA samples were analysed by targeted sequencing; 37 of the 40 samples passed library QC thresholds (Supplementary Fig. 7). Thirty-two (86.4%) samples (13 palliative patients and 19 curative intent patients) had one or more high-frequency mutations (SNV VAF > 5%) detected in tumour DNA (Fig. 2 and Supplementary Fig. 7). *TP53* was the most frequently mutated gene, with mutations detected in 30 of 37 patients (81.1%). A total of 36 different high-frequency mutations were identified across all samples and of these, ddPCR assays were available for 29 (78.4%) (Supplementary Figs. 2, 3 and 4). Sequencing results were orthogonally validated by ddPCR (Supplementary Fig. 8a), showing excellent VAF concordance in tumour DNA (R^2^ = 0.978, *p* < 0.001).

Thirty-six of the 37 tumour samples analysed by targeted NGS had sufficient DNA yield for NanoString nCounter^®^ analysis (Supplementary Fig. 9). Thirty-three samples (91.6%) showed at least one gene CNA via Nanostring nCounter^®^ analysis. Nanostring CNA data are presented in Fig. 2 as either copy number gain (CN ≥ 3), higher level amplification (CN ≥ 4) or shallow loss (CN < 1.5). Six genes were recurrently amplified: *ERBB2, VEGFA, CCNE1, KRAS, MYC* and *CCND1*, and results were validated by qPCR for the four most commonly amplified genes (*ERBB2, MYC, CCNE1* and *KRAS*). This showed excellent concordance between the two methods (R^2^ = 0.984, *p* < 0.001) (Supplementary Fig. 8b). Only patients with tumour CNA amplification (CNA^amp^) were selected for plasma cfDNA analysis by qPCR. Genes showing CN < 4, or shallow loss, were excluded because background DNA from healthy normal cells, which is inherent in plasma cfDNA analysis, impedes detection of ctDNA with a low copy number change.^35^

### ctDNA detection

Plasma cfDNA was detected in all patient blood samples although the correlation between plasma cfDNA and ctDNA levels was weak (R^2^ = 0.1846, *p* < 0.0001) (Supplementary Fig. 10a, b). Thirty-five of the 40 patients (87.5%) had one or more SNV/CNA^amp^ identified in tumour DNA suitable for investigation in plasma cfDNA, comprising 13 of 17 palliative patients and 22 of 23 curative patients. To detect low-frequency plasma cfDNA SNVs, mutation-specific ddPCR assays were used (VAF limit 0.1%^40^). When no ddPCR assay was available (three patients), the NGS panel was used (Supplementary Fig. 10a). The lower level of sensitivity of the panel for plasma cfDNA variants was determined to be a VAF of 2% by comparison with matched mutation-specific ddPCR assays. CNA^amp^ was analysed in plasma cfDNA by qPCR (Supplementary Fig. 11).

### Palliative patients

**Fig. 3:**
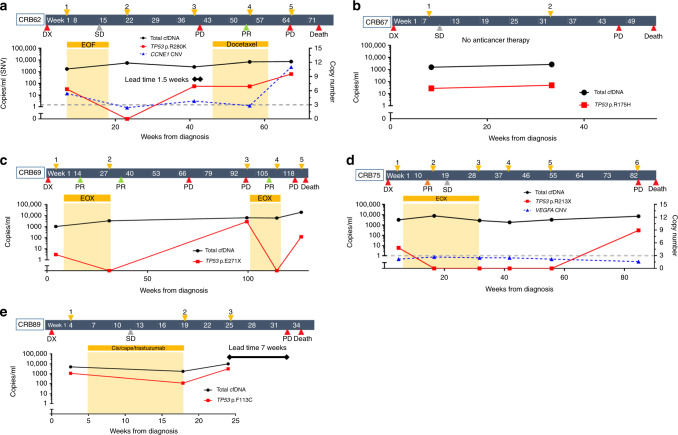
Treatment timeline for five palliative patients, showing ctDNA monitoring. Timeline shows, time of blood draws; upper arrows, CT reports; lower arrows (Dx = diagnostic CT, SD = stable disease on CT, PD = progressive disease on CT, PR = partial response on CT). Time of chemotherapy; bars (EOF = Epirubicin, oxaliplatin, 5-FU; EOX = Epirubicin, oxaliplatin, capecitabine; Cis = cisplatin, cape = capecitabine). Black line = plasma cfDNA in copies/ml (left *X*-axis). ctDNA detection: solid lines = SNV detection in copies/ml (left *X*-axis); dashed line = copy number (right *X*-axis) measured by qPCR, with threshold of 3 for amplification shown as horizontal dotted grey line.

Nine of the 13 (69.2%) palliative patients that were monitored through personalised SNV and/or CNA^amp^ assays had ctDNA detected in plasma (Supplementary Fig. 12). Serial monitoring was possible in five of the nine patients with detectable ctDNA (Fig. 3). Clinical progression was observed in all five cases, and bloods taken at the time of progressive disease showed increasing ctDNA levels, defined as >10-fold increase in ctDNA or change from negative to positive (as measured by SNV), or an increase in CN of 1. Blood samples taken at or shortly after disease response showed falling levels, using the opposing criteria to rising ctDNA levels. In contrast total plasma cfDNA levels showed a smaller degree of fluctuation. In two patients (Fig. 3a, e) a lead time of 1.5 and 7 weeks (10 and 49 days) was defined between rising ctDNA levels and clinical disease progression. Three patients showed transitory resolution of ctDNA following palliative chemotherapy and overall survival was shortest for the one patient (CRB89, Fig. 3e) who did not show resolution of ctDNA. In one case (CRB62, Fig. 3a) both SNV and CNA^amp^ were detected in plasma cfDNA and levels fluctuated synchronously with disease response and progression. In another case (CRB75, Fig. 3d) SNVs fluctuated with disease response but the CNA^amp^ was undetectable.

### Curative patients

**Fig. 4:**
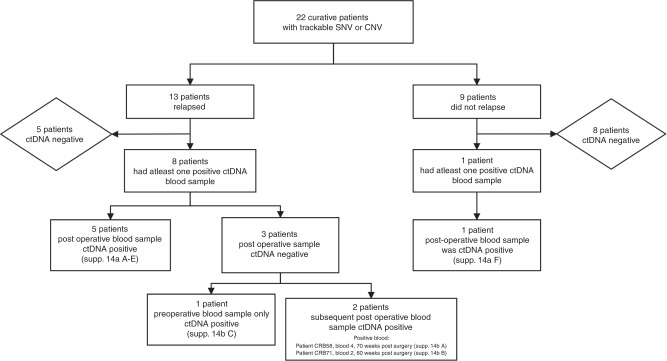
Consort diagram of the distribution of patients within the curative patient cohort with ctDNA-positive/negative blood samples. Includes reference to timelines for curative patients shown in Supplementary Fig. 14.

**Fig. 5:**
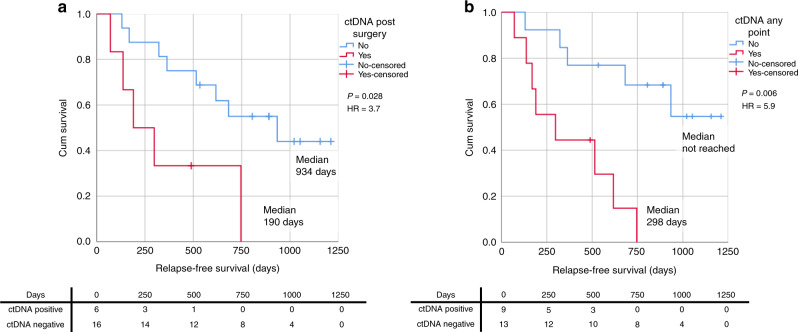
Survival in patients receiving surgery with curative intent, in days from surgery. **a** RFS in patients with ctDNA detectable in the postoperative blood draw. ctDNA measured by ddPCR (SNV) and qPCR (CNA^amp^). **b** RFS in patients with ctDNA detectable at any time-point prior to relapse.

Thirteen of the 22 curative patients that were monitored through personalised SNV and/or CNA^amp^ assays relapsed during follow-up. Eight of these patients (40.9%) had the tumour-specific variant detected in plasma cfDNA, indicating the presence of ctDNA (Supplementary Fig. 13). Of the eight patients with detectable ctDNA, five had ctDNA detected in the initial postsurgical blood, one patient had ctDNA detectable presurgery only and two patients had detectable ctDNA at a time-point after the first postsurgical blood, but before clinical relapse, at 60- and 70-weeks postsurgery (421 and 493 days) (Fig. 4 and Supplementary Fig. 14). Of the nine patients who did not relapse only one patient had ctDNA detected in their first postoperative sample, and all other samples were negative. Relapse-free survival (RFS) was significantly shorter in patients with a ctDNA-positive postsurgical blood (*n* = 6); median RFS 190 vs 934 days, HR 3.7, *p* = 0.028 (Fig. 5a). There was also a significantly shorter RFS in the patients with ctDNA-positive bloods at any time-point prior to relapse (*n* = 9), median RFS 298 days vs not reached, HR 5.9, *P* = 0.006 (Fig. 5b).

Five of the six patients with a ctDNA-positive postsurgical blood test relapsed (Fig. 4 and Supplementary Fig. 14). In four (Supplementary Fig. 14a A–D) a lead time between the positive postsurgical blood test and relapse could be identified, range 6–94 weeks (44–654 days). In the fifth patient relapse occurred before the postsurgical blood test (Supplementary Fig. 14a E). A sixth patient (Supplementary Fig. 14a F) had positive ctDNA in the postsurgical blood and had not relapsed at end of follow-up. A further blood test for this patient 231 days later was ctDNA negative.

The positive predictive value of relapse for ctDNA-positive postsurgical blood samples was high (0.83), with five out of six patients relapsing during follow-up and the negative predictive value was 0.50 (eight of the 16 patients with a negative postsurgical blood sample relapsed).

### Prognostic significance of ctDNA

**Fig. 6:**
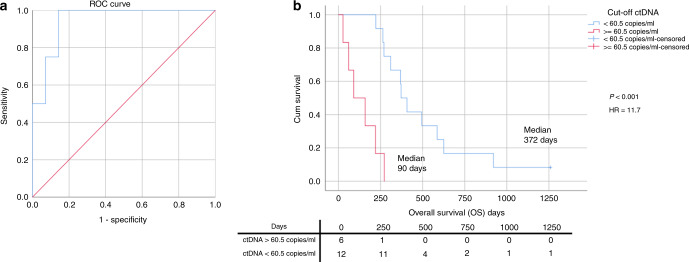
Overall survival in GOA patients with metastatic disease and a trackable SNV. **a** ROC curve to determine best cut-off point for OS less than 6 months. Using ctDNA levels (copies/ml) at diagnosis of metastatic disease. **b** Kaplan–Meier plot showing the OS for metastatic/relapse patients based on initial ctDNA levels (dichotomised by ROC-determined cut-off value). Censored: still alive at the end of follow-up.

The presence of ctDNA was a poor prognostic sign with ctDNA levels being highest in all patients towards the end of life. In total 18 patients had blood tests available within 28 days of diagnosis of metastatic disease and had a trackable SNV (tumour SNV > 5% VAF) allowing precise ctDNA quantification (13 palliative patients and five relapsed curative patients). Higher ctDNA levels correlated with shorter OS (R^2^ = 0.519, *p* = 0.005, Supplementary Fig. 15), and therefore, an optimal cut-off point for OS less than 6 months was determined using ROC analysis. The optimal value was 60.5 copies/ml, Area Under the Curve (AUC) of 0.946 (*P* = 0.008, 95% CI 0.843–1.000), sensitivity 100% and specificity 85.7% (Fig. 6a). Analysis included those patients who were ‘confirmed ctDNA negative’ (defined as presence of a trackable tumour-specific SNV but without detectable ctDNA) at the point of diagnosis of metastatic disease (Supplementary Fig. 16). Above the cut-off value of 60.5 copies/ml, the median OS was 90 days (~3 months), whereas below it the median OS was 372 days (~12.5 months) (*P* < 0.001, HR 11.7) (Fig. 6b), indicating the poor prognostic significance of high ctDNA levels.

## Discussion

Using a six-gene sequencing panel developed for GOA tumour analysis and NanoString nCounter^®^, it was possible to detect high-frequency SNVs and CNA^amp^ in 35 of 40 patient tumours. Using a personalised approach to monitor alterations in plasma cfDNA, ctDNA was detected in 18 of these 35 patients, demonstrating the utility of screening plasma cfDNA for tumour-specific variants as shown previously.^22,24^ This study shows that ctDNA levels track disease response and may predict short OS in metastatic patients, while ctDNA positivity predicts clinical relapse and short RFS in patients treated with curative intent.

Several of the tumours showed an SNV VAF > 50% (Supplementary Fig. 7). These high VAFs were measured by both NGS and ddPCR indicating that they are reflective of the true findings for these tumours. One mechanism for a high VAF maybe loss of heterozygosity (LOH) in the tumour.^41^ In keeping with this, a number of tumours have borderline evidence for shallow loss of *TP53* in the nanostring nCounter data (CRB59: *TP53* Tumour VAF 62.2%, CN 1.5 and CRB67: *TP53* Tumour VAF 54.2, CN 1.5 (Supplementary Fig. 9)). An additional mechanism may be amplification of *TP53* and those patients with the highest *TP53* VAFs appear to have gain (CN~3) of *TP53* (CRB55, CRB137 and CRB144, (Supplementary Fig. 9)).

SNVs were detectable in the plasma cfDNA of 16 patients and CNA^amp^ in six patients, with four patients having plasma cfDNA positive for both alterations. In patients where both SNV and CNA^amp^ was present in tumour tissue, the plasma cfDNA was not always positive for both variants. This may be due to tumour heterogeneity, or the lower sensitivity of CNA^amp^ detection, which requires ~10% ctDNA to gain a positive result.^35^ However, the difference in ability to detect CNA^amp^ may also reflect differences in the CN of the amplified gene in the primary tumour; with lower levels of amplification being more difficult to detect in plasma cfDNA due to a high background of DNA from healthy normal cells. In contrast SNV analysis via ddPCR could detect levels of ctDNA <0.1% VAF from 20 ng total plasma cfDNA.^40^

The described utility of tumour-specific SNV/CNA^amp^ monitoring was seen despite the presence of high genetic heterogeneity seen in GOA,^17,26^ suggesting that the majority of tracked variants were truncal. Two cases of tumour-ctDNA discordance were detected, where ctDNA was detected but at least one tumour-specific variant was absent (Supplementary Fig. 14a A and D), indicating that discordance can occur between tumour and ctDNA in GOA as noted previously.^22,25,26,42,43^

In this study, inability to detect ctDNA occurred either because of lack of a tumour-specific variant to track, or subsequent absence of ctDNA detection via plasma cfDNA analysis termed ‘confirmed ctDNA negativity’. Patients falling into either category cannot be monitored using the methods outlined in this study and in total 22 of 40 patients (55%) had no detectable ctDNA due to a combination of these reasons. Therefore, ctDNA analysis is limited to approximately half of the GOA population. Patients that had no detectable SNV or CNA^amp^ in tumour DNA may have had truncal variants that were not covered by the targeted panels utilised. Larger panels or whole-exome/genome sequencing would help identify these variants. Although these methods may improve detection of trackable variants, tumour sequencing studies (Supplementary Fig. 1) and similar analyses of ctDNA^23,25,26^ suggest the number of additional patients identified by such means maybe limited. Clinically, patients with confirmed ctDNA negativity at the diagnosis of metastatic disease is informative. Such patients were classified in the low ctDNA category (<60.5 copies/ml) in this study (Fig. 6), and as such were demonstrated to have longer OS than patients with high ctDNA levels.

The positive predictive value of relapse for ctDNA-positive postsurgical blood samples was high (0.83) and detectable ctDNA levels preceded relapse with a lead time of 6–94 weeks (44–654 days). This long potential lead time between positive ctDNA samples and clinical relapse on imaging, has been seen in other cancers.^16^ The presence of ctDNA postsurgery and prerelapse, which can be termed ‘molecular relapse’, indicates detection of minimal residual disease as the variants are tumour-specific and therefore must be derived from residual tumour cells. This is reflected in the high clinical relapse rate of ctDNA-positive patients. The described lead time between a ctDNA defined ‘molecular relapse’ postsurgery and subsequent overt clinical relapse, provides a potential therapeutic window for further anticancer interventions to target minimal residual disease.

Previous research has shown that ctDNA detection at time of primary staging is indicative of postsurgical recurrence.^23,26^ Blood samples at the time of staging were available for eight patients (Supplementary Fig. 17). The two patients (CRB105 (Supplementary Fig. 14b C), and CRB116 (Supplementary Fig. 14a B)) that were ctDNA positive at the time of staging relapsed (100%) at a median of 153 days postsurgery, whereas three of the six ctDNA-negative patients relapsed (50.0%) a median of 713 days postsurgery, thereby supporting this finding.

In two patients ctDNA became detectable prerelapse but after the initial postsurgery blood sample. This indicates that minimal residual disease can be present without detection of ctDNA, even with the highest sensitivity detection methods available (SNV ddPCR). Further support for this can be seen in metastatic patients where ctDNA-negative blood samples follow effective treatment, but relapse is still inevitable. Therefore, ctDNA-negative status is not synonymous with absence of disease and this finding should be considered in the design of future clinical studies.

Previous studies have focused on ctDNA analysis in subgroups of GOA such as HER2-positive disease^25,44^ or advanced/metastatic patients.^18,22,27,45^ One previous study^23^ used high-sensitivity SNV ddPCR analysis of ctDNA in curative patients as here; however, this did not include longitudinal monitoring. Another study used the commercial guardant360 ctDNA test across a large number of GOA patients with a smaller subset (<30 patients) of neoadjuvant and adjuvant patients where the prognostic significance of ctDNA was outlined.^26^ Our study combines high-sensitivity detection of SNV and CNA^amp^ in ctDNA with longitudinal monitoring and demonstrates that tracking individual high-frequency tumour-specific alterations has significant promise as an emerging monitoring tool. This study has shown that accurate ctDNA measurement at diagnosis of metastatic disease allows prognostication of overall survival. It has also shown that presence of ctDNA postsurgery predicts relapse in the curative setting. In addition, results of qPCR and ddPCR can be obtained within 1–2 weeks of blood sampling for patients with known tumour variants, providing rapid, and sensitive detection of ctDNA.

The main limitation of this exploratory study is the small sample size. This sample size precludes replication of ROC analysis of ctDNA levels in metastatic patients, which needs confirmation in a new/larger cohort. The significance of positive ctDNA and RFS in curative patients is also tempered by small sample size, although this result is confirmatory of similar findings in previous similar sized studies of patients undergoing treatment with curative intent.^23,26^

Overall survival rates for GOA remain poor and there has been limited improvement in treatment and survival over the last 20 years. In the palliative setting EOX (Epirubicin, Oxaliplatin, Capecitabine) has shown to be superior.^5^ While in the neoadjuvant setting treatment options include perioperative ECX^8^ (Epirubicin, Cisplatin, Capecitabine), FLOT^9^ (5-FU, Leucovorin, Oxaliplatin, Docetaxel) or chemoradiotherapy.^7^ However even in neoadjuvant studies, 5-year OS remains less than 40%. Differentiating responders from non-responders during neoadjuvant treatment is an important area of research, as delivery of postsurgical adjuvant treatment is difficult^8,9^ and some patients fail to respond to treatment. Correctly identifying non-responders would allow curtailment of ineffective neoadjuvant treatment and/or switching to alternative treatment. Plasma ctDNA has been shown to track treatment response in metastatic patients both in this study and others^22,24,25,27,44–46^ and there is also evidence that ctDNA may track response to neoadjuvant treatment in a similar way.^23,26^ Indeed, in this study a fall in ctDNA in response to neoadjuvant chemotherapy was seen in two patients (Supplementary Fig. 14a B and C) indicating a response to treatment. The use of ctDNA to monitor response to neoadjuvant therapy has precedence in other cancers; in breast cancer a rapid decrease in ctDNA after neoadjuvant chemotherapy has been associated with a pathological complete response^47^ and conversely a slow decrease in ctDNA was associated with short survival;^48^ whereas in rectal cancer worse recurrence free survival was seen if ctDNA was detectable after neoadjuvant chemoradiotherapy.^49^ Therefore, ctDNA tracking during neoadjuvant treatment of GOA may be feasible, allowing monitoring of response in future trials, with the option of stopping treatment or switching to an alternative regimen if ctDNA levels do not fall.

As a result of the developments in ctDNA research, ctDNA analysis is included in many current GOA trials and plays a role in treatment selection.^50–52^ One important aim is to determine if further anticancer therapy in preclinical relapse, ctDNA-positive (molecular relapse) patients, can alter the trajectory of disease. Confirmation and integration of the findings of this study into clinical trials is essential to define the clinical utility of ctDNA and improve the outcomes for patients with GOA.

### Supplementary information

All supplementary Data

## Data Availability

Data are available in supplementary data files attached. Sequencing data readouts can be requested from the corresponding author.
