# Supplementary material for: Longitudinal monitoring of circulating tumour DNA improves prognostication and relapse detection in gastroesophageal adenocarcinoma
Source: Br J Cancer. 2020 Jul 28;123(8):1271–9. doi: 10.1038/s41416-020-1002-8 (PMC7555811; doi:10.1038/s41416-020-1002-8)
Supplement: Supplementary file 1 — All supplementary Data [file 41416_2020_1002_MOESM1_ESM.pptx]

## Slide 1
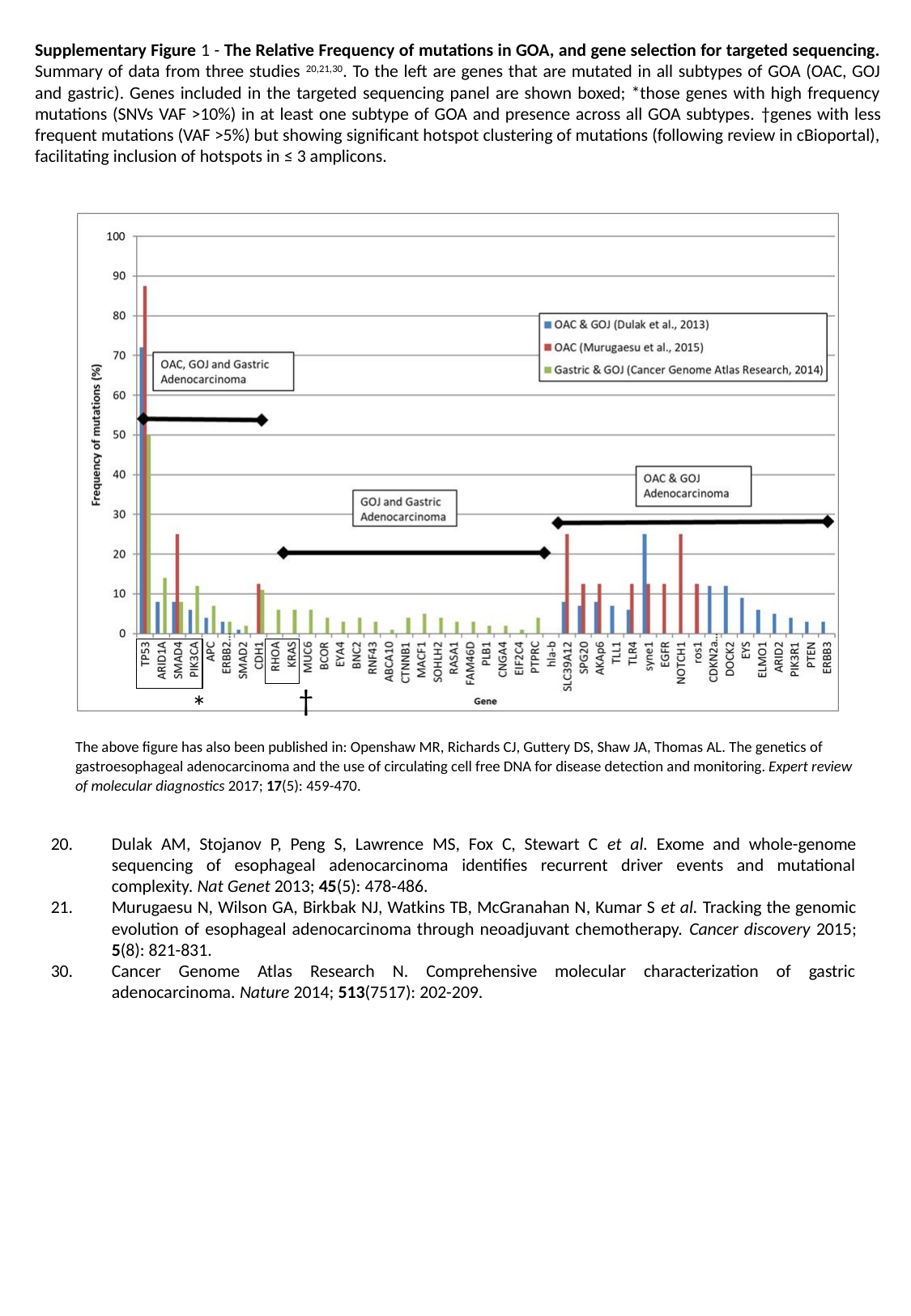

Supplementary Figure 1 - The Relative Frequency of mutations in GOA, and gene selection for targeted sequencing. Summary of data from three studies 20,21,30. To the left are genes that are mutated in all subtypes of GOA (OAC, GOJ and gastric). Genes included in the targeted sequencing panel are shown boxed; *those genes with high frequency mutations (SNVs VAF >10%) in at least one subtype of GOA and presence across all GOA subtypes. †genes with less frequent mutations (VAF >5%) but showing significant hotspot clustering of mutations (following review in cBioportal), facilitating inclusion of hotspots in ≤ 3 amplicons.
†
*
The above figure has also been published in: Openshaw MR, Richards CJ, Guttery DS, Shaw JA, Thomas AL. The genetics of gastroesophageal adenocarcinoma and the use of circulating cell free DNA for disease detection and monitoring. Expert review of molecular diagnostics 2017; 17(5): 459-470.
20.	Dulak AM, Stojanov P, Peng S, Lawrence MS, Fox C, Stewart C et al. Exome and whole-genome sequencing of esophageal adenocarcinoma identifies recurrent driver events and mutational complexity. Nat Genet 2013; 45(5): 478-486.
21.	Murugaesu N, Wilson GA, Birkbak NJ, Watkins TB, McGranahan N, Kumar S et al. Tracking the genomic evolution of esophageal adenocarcinoma through neoadjuvant chemotherapy. Cancer discovery 2015; 5(8): 821-831.
30.	Cancer Genome Atlas Research N. Comprehensive molecular characterization of gastric adenocarcinoma. Nature 2014; 513(7517): 202-209.

## Slide 2
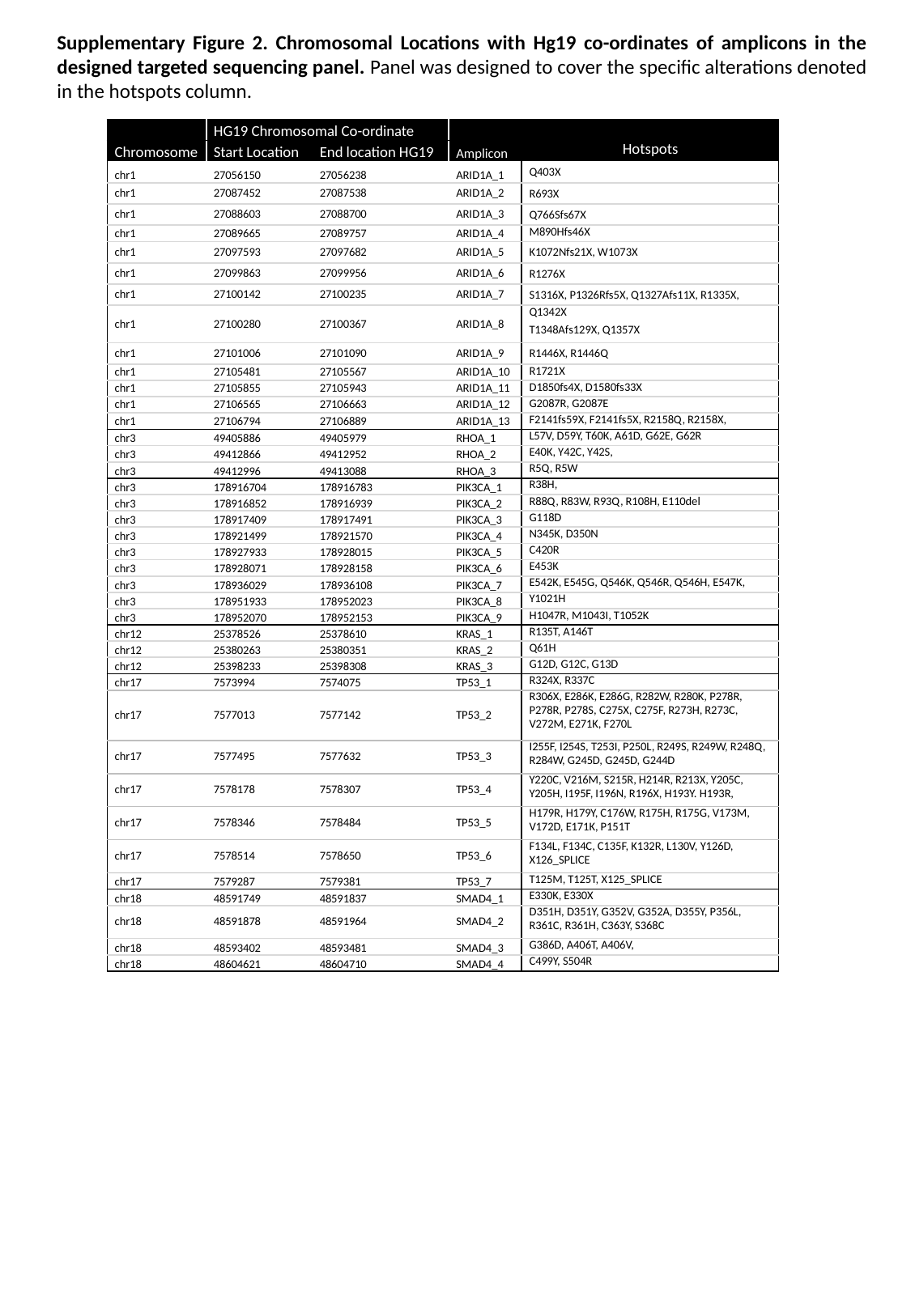

Supplementary Figure 2. Chromosomal Locations with Hg19 co-ordinates of amplicons in the designed targeted sequencing panel. Panel was designed to cover the specific alterations denoted in the hotspots column.
| | HG19 Chromosomal Co-ordinate | | | |
| --- | --- | --- | --- | --- |
| Chromosome | Start Location | End location HG19 | Amplicon | Hotspots |
| chr1 | 27056150 | 27056238 | ARID1A\_1 | Q403X |
| chr1 | 27087452 | 27087538 | ARID1A\_2 | R693X |
| chr1 | 27088603 | 27088700 | ARID1A\_3 | Q766Sfs67X |
| chr1 | 27089665 | 27089757 | ARID1A\_4 | M890Hfs46X |
| chr1 | 27097593 | 27097682 | ARID1A\_5 | K1072Nfs21X, W1073X |
| chr1 | 27099863 | 27099956 | ARID1A\_6 | R1276X |
| chr1 | 27100142 | 27100235 | ARID1A\_7 | S1316X, P1326Rfs5X, Q1327Afs11X, R1335X, |
| chr1 | 27100280 | 27100367 | ARID1A\_8 | Q1342X T1348Afs129X, Q1357X |
| chr1 | 27101006 | 27101090 | ARID1A\_9 | R1446X, R1446Q |
| chr1 | 27105481 | 27105567 | ARID1A\_10 | R1721X |
| chr1 | 27105855 | 27105943 | ARID1A\_11 | D1850fs4X, D1580fs33X |
| chr1 | 27106565 | 27106663 | ARID1A\_12 | G2087R, G2087E |
| chr1 | 27106794 | 27106889 | ARID1A\_13 | F2141fs59X, F2141fs5X, R2158Q, R2158X, |
| chr3 | 49405886 | 49405979 | RHOA\_1 | L57V, D59Y, T60K, A61D, G62E, G62R |
| chr3 | 49412866 | 49412952 | RHOA\_2 | E40K, Y42C, Y42S, |
| chr3 | 49412996 | 49413088 | RHOA\_3 | R5Q, R5W |
| chr3 | 178916704 | 178916783 | PIK3CA\_1 | R38H, |
| chr3 | 178916852 | 178916939 | PIK3CA\_2 | R88Q, R83W, R93Q, R108H, E110del |
| chr3 | 178917409 | 178917491 | PIK3CA\_3 | G118D |
| chr3 | 178921499 | 178921570 | PIK3CA\_4 | N345K, D350N |
| chr3 | 178927933 | 178928015 | PIK3CA\_5 | C420R |
| chr3 | 178928071 | 178928158 | PIK3CA\_6 | E453K |
| chr3 | 178936029 | 178936108 | PIK3CA\_7 | E542K, E545G, Q546K, Q546R, Q546H, E547K, |
| chr3 | 178951933 | 178952023 | PIK3CA\_8 | Y1021H |
| chr3 | 178952070 | 178952153 | PIK3CA\_9 | H1047R, M1043I, T1052K |
| chr12 | 25378526 | 25378610 | KRAS\_1 | R135T, A146T |
| chr12 | 25380263 | 25380351 | KRAS\_2 | Q61H |
| chr12 | 25398233 | 25398308 | KRAS\_3 | G12D, G12C, G13D |
| chr17 | 7573994 | 7574075 | TP53\_1 | R324X, R337C |
| chr17 | 7577013 | 7577142 | TP53\_2 | R306X, E286K, E286G, R282W, R280K, P278R, P278R, P278S, C275X, C275F, R273H, R273C, V272M, E271K, F270L |
| chr17 | 7577495 | 7577632 | TP53\_3 | I255F, I254S, T253I, P250L, R249S, R249W, R248Q, R284W, G245D, G245D, G244D |
| chr17 | 7578178 | 7578307 | TP53\_4 | Y220C, V216M, S215R, H214R, R213X, Y205C, Y205H, I195F, I196N, R196X, H193Y. H193R, |
| chr17 | 7578346 | 7578484 | TP53\_5 | H179R, H179Y, C176W, R175H, R175G, V173M, V172D, E171K, P151T |
| chr17 | 7578514 | 7578650 | TP53\_6 | F134L, F134C, C135F, K132R, L130V, Y126D, X126\_SPLICE |
| chr17 | 7579287 | 7579381 | TP53\_7 | T125M, T125T, X125\_SPLICE |
| chr18 | 48591749 | 48591837 | SMAD4\_1 | E330K, E330X |
| chr18 | 48591878 | 48591964 | SMAD4\_2 | D351H, D351Y, G352V, G352A, D355Y, P356L, R361C, R361H, C363Y, S368C |
| chr18 | 48593402 | 48593481 | SMAD4\_3 | G386D, A406T, A406V, |
| chr18 | 48604621 | 48604710 | SMAD4\_4 | C499Y, S504R |

## Slide 3
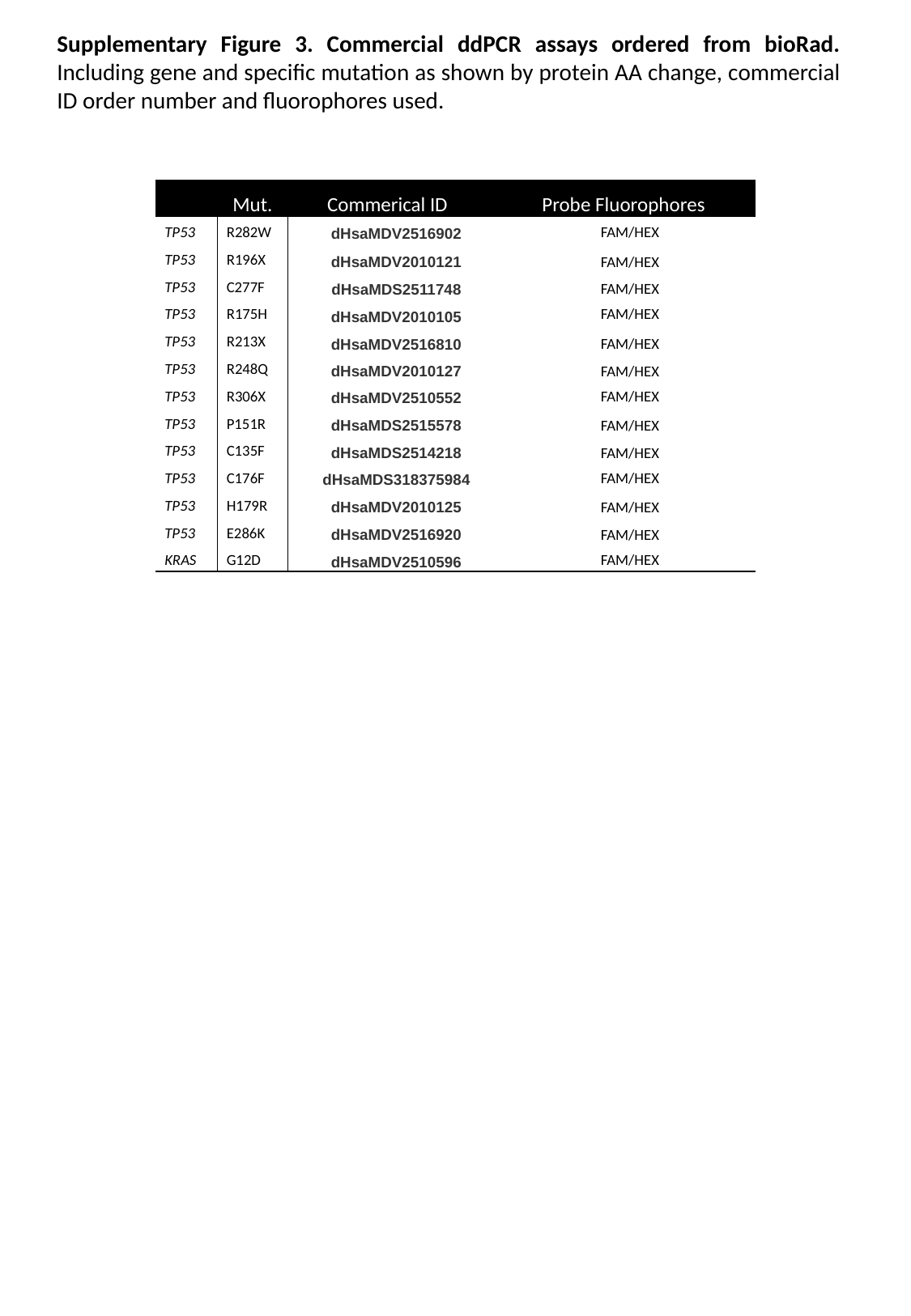

Supplementary Figure 3. Commercial ddPCR assays ordered from bioRad. Including gene and specific mutation as shown by protein AA change, commercial ID order number and fluorophores used.
| Gene | Mut. | Commerical ID | Probe Fluorophores | |
| --- | --- | --- | --- | --- |
| TP53 | R282W | dHsaMDV2516902 | | FAM/HEX |
| TP53 | R196X | dHsaMDV2010121 | | FAM/HEX |
| TP53 | C277F | dHsaMDS2511748 | | FAM/HEX |
| TP53 | R175H | dHsaMDV2010105 | | FAM/HEX |
| TP53 | R213X | dHsaMDV2516810 | | FAM/HEX |
| TP53 | R248Q | dHsaMDV2010127 | | FAM/HEX |
| TP53 | R306X | dHsaMDV2510552 | | FAM/HEX |
| TP53 | P151R | dHsaMDS2515578 | | FAM/HEX |
| TP53 | C135F | dHsaMDS2514218 | | FAM/HEX |
| TP53 | C176F | dHsaMDS318375984 | | FAM/HEX |
| TP53 | H179R | dHsaMDV2010125 | | FAM/HEX |
| TP53 | E286K | dHsaMDV2516920 | | FAM/HEX |
| KRAS | G12D | dHsaMDV2510596 | | FAM/HEX |

## Slide 4
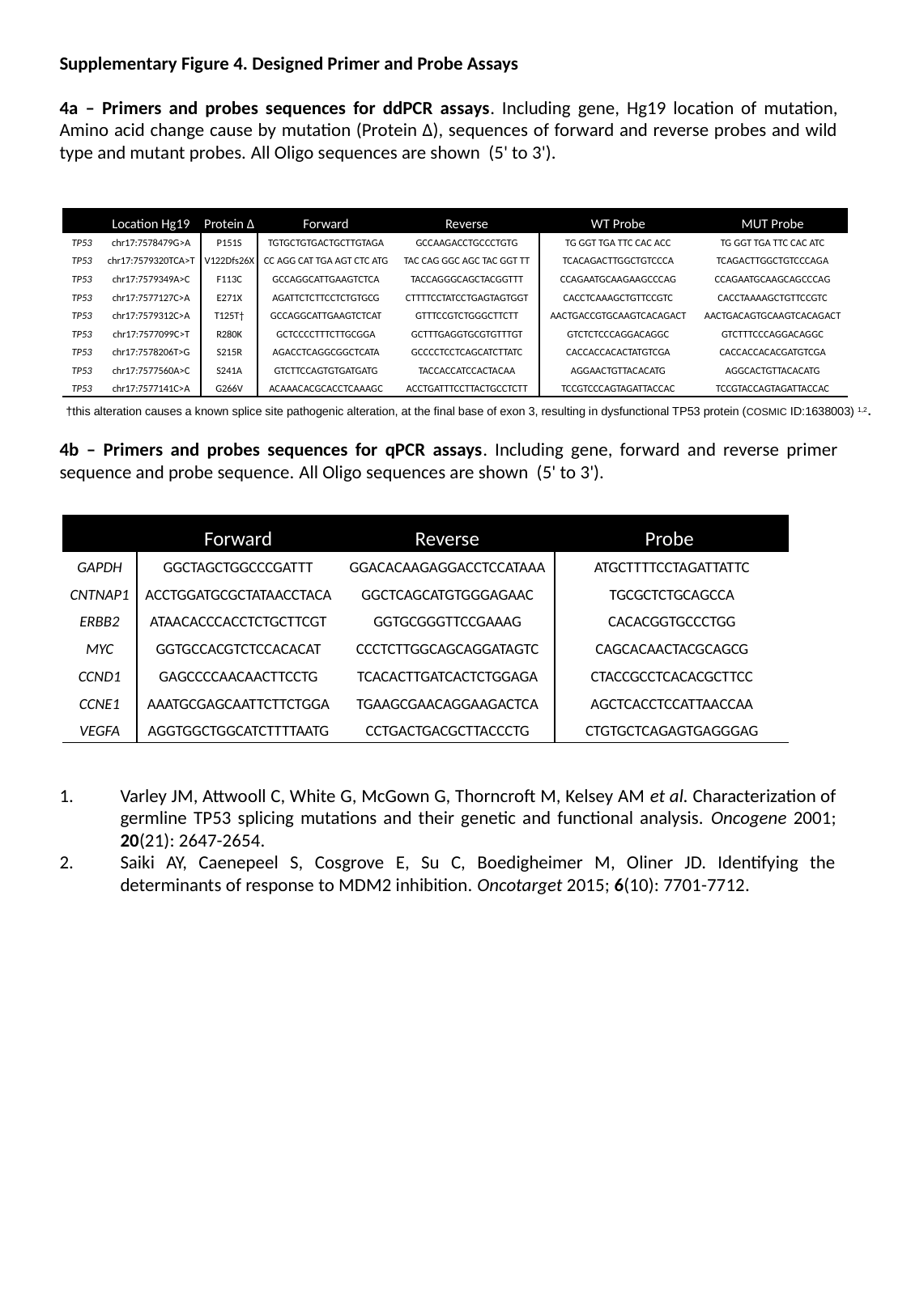

Supplementary Figure 4. Designed Primer and Probe Assays
4a – Primers and probes sequences for ddPCR assays. Including gene, Hg19 location of mutation, Amino acid change cause by mutation (Protein Δ), sequences of forward and reverse probes and wild type and mutant probes. All Oligo sequences are shown (5' to 3').
| Gene | Location Hg19 | Protein Δ | Forward | Reverse | WT Probe | MUT Probe |
| --- | --- | --- | --- | --- | --- | --- |
| TP53 | chr17:7578479G>A | P151S | TGTGCTGTGACTGCTTGTAGA | GCCAAGACCTGCCCTGTG | TG GGT TGA TTC CAC ACC | TG GGT TGA TTC CAC ATC |
| TP53 | chr17:7579320TCA>T | V122Dfs26X | CC AGG CAT TGA AGT CTC ATG | TAC CAG GGC AGC TAC GGT TT | TCACAGACTTGGCTGTCCCA | TCAGACTTGGCTGTCCCAGA |
| TP53 | chr17:7579349A>C | F113C | GCCAGGCATTGAAGTCTCA | TACCAGGGCAGCTACGGTTT | CCAGAATGCAAGAAGCCCAG | CCAGAATGCAAGCAGCCCAG |
| TP53 | chr17:7577127C>A | E271X | AGATTCTCTTCCTCTGTGCG | CTTTTCCTATCCTGAGTAGTGGT | CACCTCAAAGCTGTTCCGTC | CACCTAAAAGCTGTTCCGTC |
| TP53 | chr17:7579312C>A | T125T† | GCCAGGCATTGAAGTCTCAT | GTTTCCGTCTGGGCTTCTT | AACTGACCGTGCAAGTCACAGACT | AACTGACAGTGCAAGTCACAGACT |
| TP53 | chr17:7577099C>T | R280K | GCTCCCCTTTCTTGCGGA | GCTTTGAGGTGCGTGTTTGT | GTCTCTCCCAGGACAGGC | GTCTTTCCCAGGACAGGC |
| TP53 | chr17:7578206T>G | S215R | AGACCTCAGGCGGCTCATA | GCCCCTCCTCAGCATCTTATC | CACCACCACACTATGTCGA | CACCACCACACGATGTCGA |
| TP53 | chr17:7577560A>C | S241A | GTCTTCCAGTGTGATGATG | TACCACCATCCACTACAA | AGGAACTGTTACACATG | AGGCACTGTTACACATG |
| TP53 | chr17:7577141C>A | G266V | ACAAACACGCACCTCAAAGC | ACCTGATTTCCTTACTGCCTCTT | TCCGTCCCAGTAGATTACCAC | TCCGTACCAGTAGATTACCAC |
†this alteration causes a known splice site pathogenic alteration, at the final base of exon 3, resulting in dysfunctional TP53 protein (COSMIC ID:1638003) 1,2.
4b – Primers and probes sequences for qPCR assays. Including gene, forward and reverse primer sequence and probe sequence. All Oligo sequences are shown (5' to 3').
| Gene | Forward | Reverse | Probe |
| --- | --- | --- | --- |
| GAPDH | GGCTAGCTGGCCCGATTT | GGACACAAGAGGACCTCCATAAA | ATGCTTTTCCTAGATTATTC |
| CNTNAP1 | ACCTGGATGCGCTATAACCTACA | GGCTCAGCATGTGGGAGAAC | TGCGCTCTGCAGCCA |
| ERBB2 | ATAACACCCACCTCTGCTTCGT | GGTGCGGGTTCCGAAAG | CACACGGTGCCCTGG |
| MYC | GGTGCCACGTCTCCACACAT | CCCTCTTGGCAGCAGGATAGTC | CAGCACAACTACGCAGCG |
| CCND1 | GAGCCCCAACAACTTCCTG | TCACACTTGATCACTCTGGAGA | CTACCGCCTCACACGCTTCC |
| CCNE1 | AAATGCGAGCAATTCTTCTGGA | TGAAGCGAACAGGAAGACTCA | AGCTCACCTCCATTAACCAA |
| VEGFA | AGGTGGCTGGCATCTTTTAATG | CCTGACTGACGCTTACCCTG | CTGTGCTCAGAGTGAGGGAG |
1.	Varley JM, Attwooll C, White G, McGown G, Thorncroft M, Kelsey AM et al. Characterization of germline TP53 splicing mutations and their genetic and functional analysis. Oncogene 2001; 20(21): 2647-2654.
2.	Saiki AY, Caenepeel S, Cosgrove E, Su C, Boedigheimer M, Oliner JD. Identifying the determinants of response to MDM2 inhibition. Oncotarget 2015; 6(10): 7701-7712.

## Slide 5
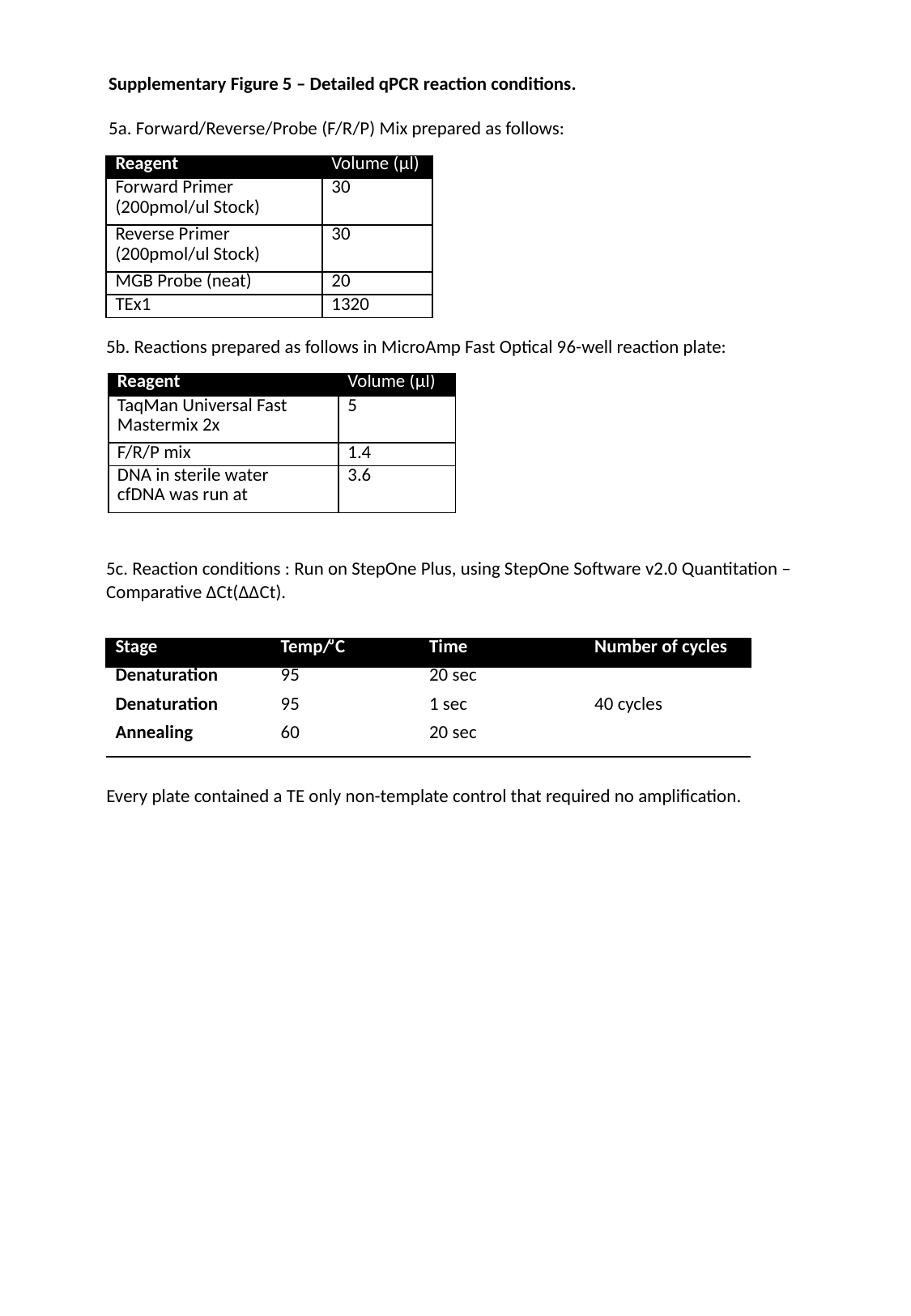

Supplementary Figure 5 – Detailed qPCR reaction conditions.
5a. Forward/Reverse/Probe (F/R/P) Mix prepared as follows:
| Reagent | Volume (µl) |
| --- | --- |
| Forward Primer (200pmol/ul Stock) | 30 |
| Reverse Primer (200pmol/ul Stock) | 30 |
| MGB Probe (neat) | 20 |
| TEx1 | 1320 |
5b. Reactions prepared as follows in MicroAmp Fast Optical 96-well reaction plate:
| Reagent | Volume (µl) |
| --- | --- |
| TaqMan Universal Fast Mastermix 2x | 5 |
| F/R/P mix | 1.4 |
| DNA in sterile water cfDNA was run at | 3.6 |
5c. Reaction conditions : Run on StepOne Plus, using StepOne Software v2.0 Quantitation – Comparative ΔCt(ΔΔCt).
| Stage | Temp/⁰C | Time | Number of cycles |
| --- | --- | --- | --- |
| Denaturation | 95 | 20 sec | |
| Denaturation | 95 | 1 sec | 40 cycles |
| Annealing | 60 | 20 sec | |
Every plate contained a TE only non-template control that required no amplification.

## Slide 6
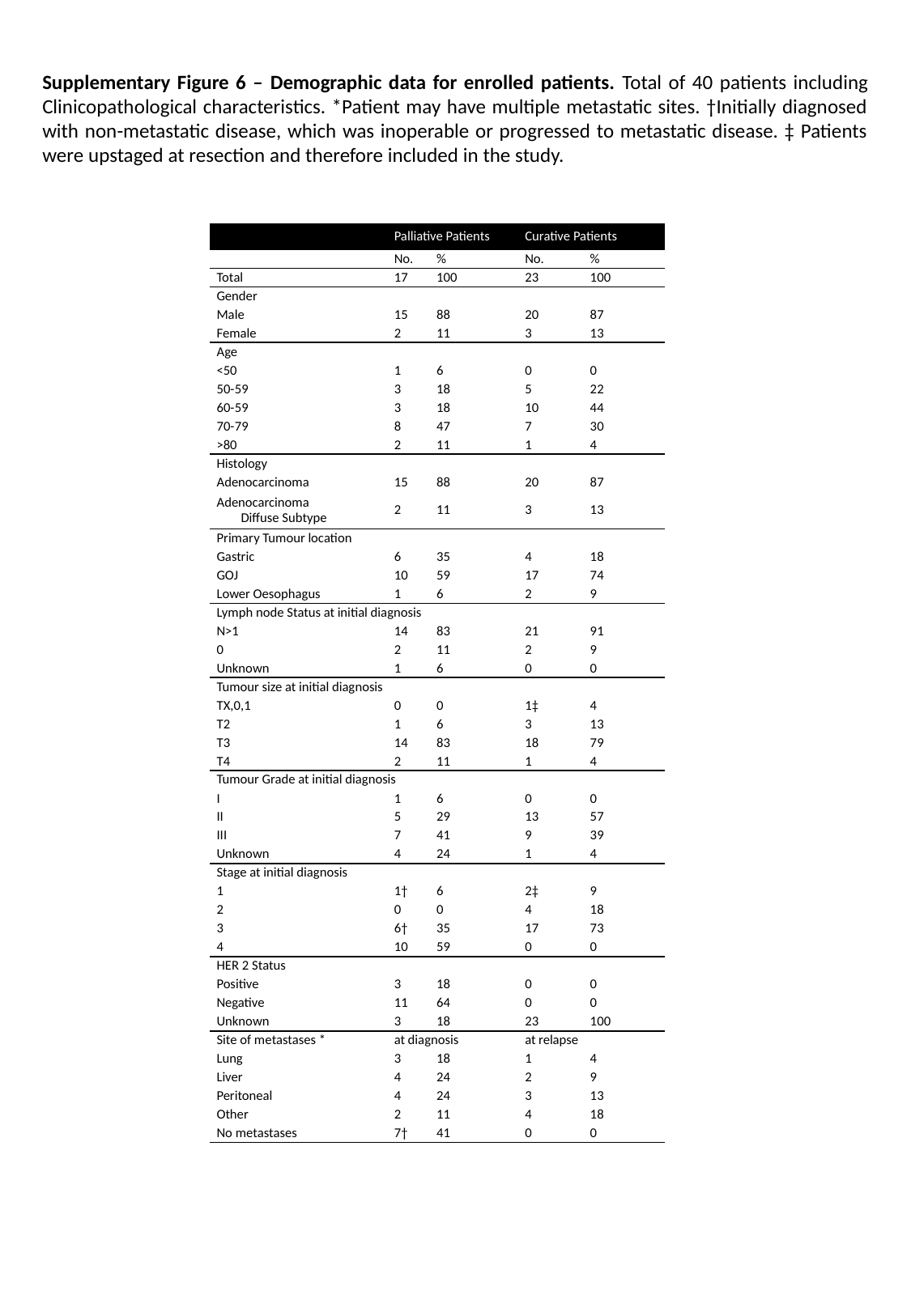

Supplementary Figure 6 – Demographic data for enrolled patients. Total of 40 patients including Clinicopathological characteristics. *Patient may have multiple metastatic sites. †Initially diagnosed with non-metastatic disease, which was inoperable or progressed to metastatic disease. ‡ Patients were upstaged at resection and therefore included in the study.
| | Palliative Patients | | Curative Patients | |
| --- | --- | --- | --- | --- |
| | No. | % | No. | % |
| Total | 17 | 100 | 23 | 100 |
| Gender | | | | |
| Male | 15 | 88 | 20 | 87 |
| Female | 2 | 11 | 3 | 13 |
| Age | | | | |
| <50 | 1 | 6 | 0 | 0 |
| 50-59 | 3 | 18 | 5 | 22 |
| 60-59 | 3 | 18 | 10 | 44 |
| 70-79 | 8 | 47 | 7 | 30 |
| >80 | 2 | 11 | 1 | 4 |
| Histology | | | | |
| Adenocarcinoma | 15 | 88 | 20 | 87 |
| Adenocarcinoma Diffuse Subtype | 2 | 11 | 3 | 13 |
| Primary Tumour location | | | | |
| Gastric | 6 | 35 | 4 | 18 |
| GOJ | 10 | 59 | 17 | 74 |
| Lower Oesophagus | 1 | 6 | 2 | 9 |
| Lymph node Status at initial diagnosis | | | | |
| N>1 | 14 | 83 | 21 | 91 |
| 0 | 2 | 11 | 2 | 9 |
| Unknown | 1 | 6 | 0 | 0 |
| Tumour size at initial diagnosis | | | | |
| TX,0,1 | 0 | 0 | 1‡ | 4 |
| T2 | 1 | 6 | 3 | 13 |
| T3 | 14 | 83 | 18 | 79 |
| T4 | 2 | 11 | 1 | 4 |
| Tumour Grade at initial diagnosis | | | | |
| I | 1 | 6 | 0 | 0 |
| II | 5 | 29 | 13 | 57 |
| III | 7 | 41 | 9 | 39 |
| Unknown | 4 | 24 | 1 | 4 |
| Stage at initial diagnosis | | | | |
| 1 | 1† | 6 | 2‡ | 9 |
| 2 | 0 | 0 | 4 | 18 |
| 3 | 6† | 35 | 17 | 73 |
| 4 | 10 | 59 | 0 | 0 |
| HER 2 Status | | | | |
| Positive | 3 | 18 | 0 | 0 |
| Negative | 11 | 64 | 0 | 0 |
| Unknown | 3 | 18 | 23 | 100 |
| Site of metastases \* | at diagnosis | | at relapse | |
| Lung | 3 | 18 | 1 | 4 |
| Liver | 4 | 24 | 2 | 9 |
| Peritoneal | 4 | 24 | 3 | 13 |
| Other | 2 | 11 | 4 | 18 |
| No metastases | 7† | 41 | 0 | 0 |

## Slide 7
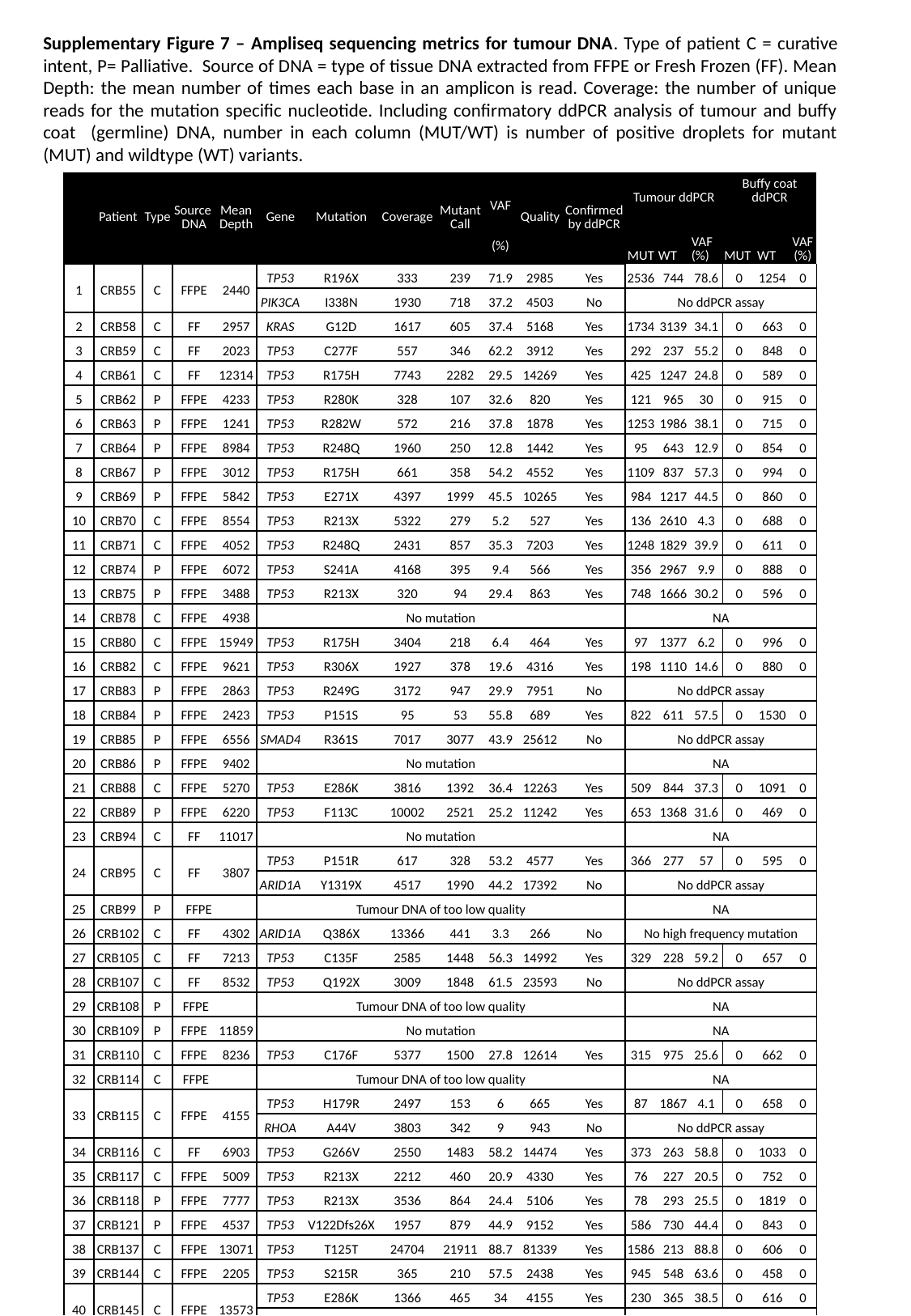

Supplementary Figure 7 – Ampliseq sequencing metrics for tumour DNA. Type of patient C = curative intent, P= Palliative. Source of DNA = type of tissue DNA extracted from FFPE or Fresh Frozen (FF). Mean Depth: the mean number of times each base in an amplicon is read. Coverage: the number of unique reads for the mutation specific nucleotide. Including confirmatory ddPCR analysis of tumour and buffy coat (germline) DNA, number in each column (MUT/WT) is number of positive droplets for mutant (MUT) and wildtype (WT) variants.
| | Patient | Type | Source DNA | Mean Depth | Gene | Mutation | Coverage | Mutant Call | VAF | Quality | Confirmed by ddPCR | Tumour ddPCR | | | Buffy coat ddPCR | | |
| --- | --- | --- | --- | --- | --- | --- | --- | --- | --- | --- | --- | --- | --- | --- | --- | --- | --- |
| | | | | | | | | | | | | MUT | WT | VAF (%) | MUT | WT | VAF (%) |
| | | | | | | | | | (%) | | | MUT | WT | VAF (%) | MUT | WT | VAF (%) |
| 1 | CRB55 | C | FFPE | 2440 | TP53 | R196X | 333 | 239 | 71.9 | 2985 | Yes | 2536 | 744 | 78.6 | 0 | 1254 | 0 |
| | | | | | PIK3CA | I338N | 1930 | 718 | 37.2 | 4503 | No | No ddPCR assay | | | | | |
| 2 | CRB58 | C | FF | 2957 | KRAS | G12D | 1617 | 605 | 37.4 | 5168 | Yes | 1734 | 3139 | 34.1 | 0 | 663 | 0 |
| 3 | CRB59 | C | FF | 2023 | TP53 | C277F | 557 | 346 | 62.2 | 3912 | Yes | 292 | 237 | 55.2 | 0 | 848 | 0 |
| 4 | CRB61 | C | FF | 12314 | TP53 | R175H | 7743 | 2282 | 29.5 | 14269 | Yes | 425 | 1247 | 24.8 | 0 | 589 | 0 |
| 5 | CRB62 | P | FFPE | 4233 | TP53 | R280K | 328 | 107 | 32.6 | 820 | Yes | 121 | 965 | 30 | 0 | 915 | 0 |
| 6 | CRB63 | P | FFPE | 1241 | TP53 | R282W | 572 | 216 | 37.8 | 1878 | Yes | 1253 | 1986 | 38.1 | 0 | 715 | 0 |
| 7 | CRB64 | P | FFPE | 8984 | TP53 | R248Q | 1960 | 250 | 12.8 | 1442 | Yes | 95 | 643 | 12.9 | 0 | 854 | 0 |
| 8 | CRB67 | P | FFPE | 3012 | TP53 | R175H | 661 | 358 | 54.2 | 4552 | Yes | 1109 | 837 | 57.3 | 0 | 994 | 0 |
| 9 | CRB69 | P | FFPE | 5842 | TP53 | E271X | 4397 | 1999 | 45.5 | 10265 | Yes | 984 | 1217 | 44.5 | 0 | 860 | 0 |
| 10 | CRB70 | C | FFPE | 8554 | TP53 | R213X | 5322 | 279 | 5.2 | 527 | Yes | 136 | 2610 | 4.3 | 0 | 688 | 0 |
| 11 | CRB71 | C | FFPE | 4052 | TP53 | R248Q | 2431 | 857 | 35.3 | 7203 | Yes | 1248 | 1829 | 39.9 | 0 | 611 | 0 |
| 12 | CRB74 | P | FFPE | 6072 | TP53 | S241A | 4168 | 395 | 9.4 | 566 | Yes | 356 | 2967 | 9.9 | 0 | 888 | 0 |
| 13 | CRB75 | P | FFPE | 3488 | TP53 | R213X | 320 | 94 | 29.4 | 863 | Yes | 748 | 1666 | 30.2 | 0 | 596 | 0 |
| 14 | CRB78 | C | FFPE | 4938 | No mutation | | | | | | | NA | | | | | |
| 15 | CRB80 | C | FFPE | 15949 | TP53 | R175H | 3404 | 218 | 6.4 | 464 | Yes | 97 | 1377 | 6.2 | 0 | 996 | 0 |
| 16 | CRB82 | C | FFPE | 9621 | TP53 | R306X | 1927 | 378 | 19.6 | 4316 | Yes | 198 | 1110 | 14.6 | 0 | 880 | 0 |
| 17 | CRB83 | P | FFPE | 2863 | TP53 | R249G | 3172 | 947 | 29.9 | 7951 | No | No ddPCR assay | | | | | |
| 18 | CRB84 | P | FFPE | 2423 | TP53 | P151S | 95 | 53 | 55.8 | 689 | Yes | 822 | 611 | 57.5 | 0 | 1530 | 0 |
| 19 | CRB85 | P | FFPE | 6556 | SMAD4 | R361S | 7017 | 3077 | 43.9 | 25612 | No | No ddPCR assay | | | | | |
| 20 | CRB86 | P | FFPE | 9402 | No mutation | | | | | | | NA | | | | | |
| 21 | CRB88 | C | FFPE | 5270 | TP53 | E286K | 3816 | 1392 | 36.4 | 12263 | Yes | 509 | 844 | 37.3 | 0 | 1091 | 0 |
| 22 | CRB89 | P | FFPE | 6220 | TP53 | F113C | 10002 | 2521 | 25.2 | 11242 | Yes | 653 | 1368 | 31.6 | 0 | 469 | 0 |
| 23 | CRB94 | C | FF | 11017 | No mutation | | | | | | | NA | | | | | |
| 24 | CRB95 | C | FF | 3807 | TP53 | P151R | 617 | 328 | 53.2 | 4577 | Yes | 366 | 277 | 57 | 0 | 595 | 0 |
| | | | | | ARID1A | Y1319X | 4517 | 1990 | 44.2 | 17392 | No | No ddPCR assay | | | | | |
| 25 | CRB99 | P | FFPE | | Tumour DNA of too low quality | | | | | | | NA | | | | | |
| 26 | CRB102 | C | FF | 4302 | ARID1A | Q386X | 13366 | 441 | 3.3 | 266 | No | No high frequency mutation | | | | | |
| 27 | CRB105 | C | FF | 7213 | TP53 | C135F | 2585 | 1448 | 56.3 | 14992 | Yes | 329 | 228 | 59.2 | 0 | 657 | 0 |
| 28 | CRB107 | C | FF | 8532 | TP53 | Q192X | 3009 | 1848 | 61.5 | 23593 | No | No ddPCR assay | | | | | |
| 29 | CRB108 | P | FFPE | | Tumour DNA of too low quality | | | | | | | NA | | | | | |
| 30 | CRB109 | P | FFPE | 11859 | No mutation | | | | | | | NA | | | | | |
| 31 | CRB110 | C | FFPE | 8236 | TP53 | C176F | 5377 | 1500 | 27.8 | 12614 | Yes | 315 | 975 | 25.6 | 0 | 662 | 0 |
| 32 | CRB114 | C | FFPE | | Tumour DNA of too low quality | | | | | | | NA | | | | | |
| 33 | CRB115 | C | FFPE | 4155 | TP53 | H179R | 2497 | 153 | 6 | 665 | Yes | 87 | 1867 | 4.1 | 0 | 658 | 0 |
| | | | | | RHOA | A44V | 3803 | 342 | 9 | 943 | No | No ddPCR assay | | | | | |
| 34 | CRB116 | C | FF | 6903 | TP53 | G266V | 2550 | 1483 | 58.2 | 14474 | Yes | 373 | 263 | 58.8 | 0 | 1033 | 0 |
| 35 | CRB117 | C | FFPE | 5009 | TP53 | R213X | 2212 | 460 | 20.9 | 4330 | Yes | 76 | 227 | 20.5 | 0 | 752 | 0 |
| 36 | CRB118 | P | FFPE | 7777 | TP53 | R213X | 3536 | 864 | 24.4 | 5106 | Yes | 78 | 293 | 25.5 | 0 | 1819 | 0 |
| 37 | CRB121 | P | FFPE | 4537 | TP53 | V122Dfs26X | 1957 | 879 | 44.9 | 9152 | Yes | 586 | 730 | 44.4 | 0 | 843 | 0 |
| 38 | CRB137 | C | FFPE | 13071 | TP53 | T125T | 24704 | 21911 | 88.7 | 81339 | Yes | 1586 | 213 | 88.8 | 0 | 606 | 0 |
| 39 | CRB144 | C | FFPE | 2205 | TP53 | S215R | 365 | 210 | 57.5 | 2438 | Yes | 945 | 548 | 63.6 | 0 | 458 | 0 |
| 40 | CRB145 | C | FFPE | 13573 | TP53 | E286K | 1366 | 465 | 34 | 4155 | Yes | 230 | 365 | 38.5 | 0 | 616 | 0 |
| | | | | | PIK3CA | E545K | 14058 | 901 | 6.4 | 896 | No | Not done | | | | | |

## Slide 8
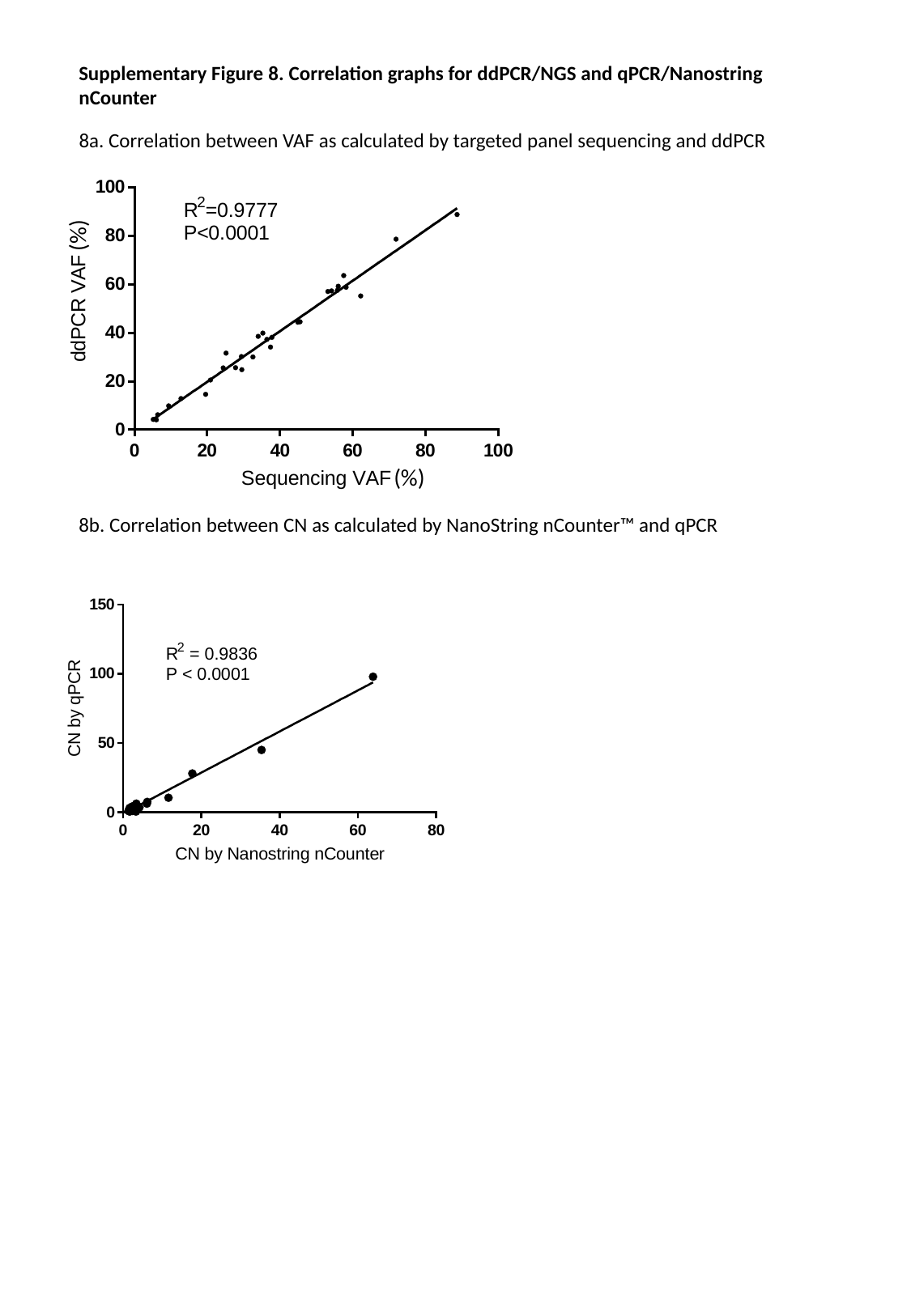

Supplementary Figure 8. Correlation graphs for ddPCR/NGS and qPCR/Nanostring nCounter
8a. Correlation between VAF as calculated by targeted panel sequencing and ddPCR
(%)
(%)
8b. Correlation between CN as calculated by NanoString nCounter™ and qPCR

## Slide 9
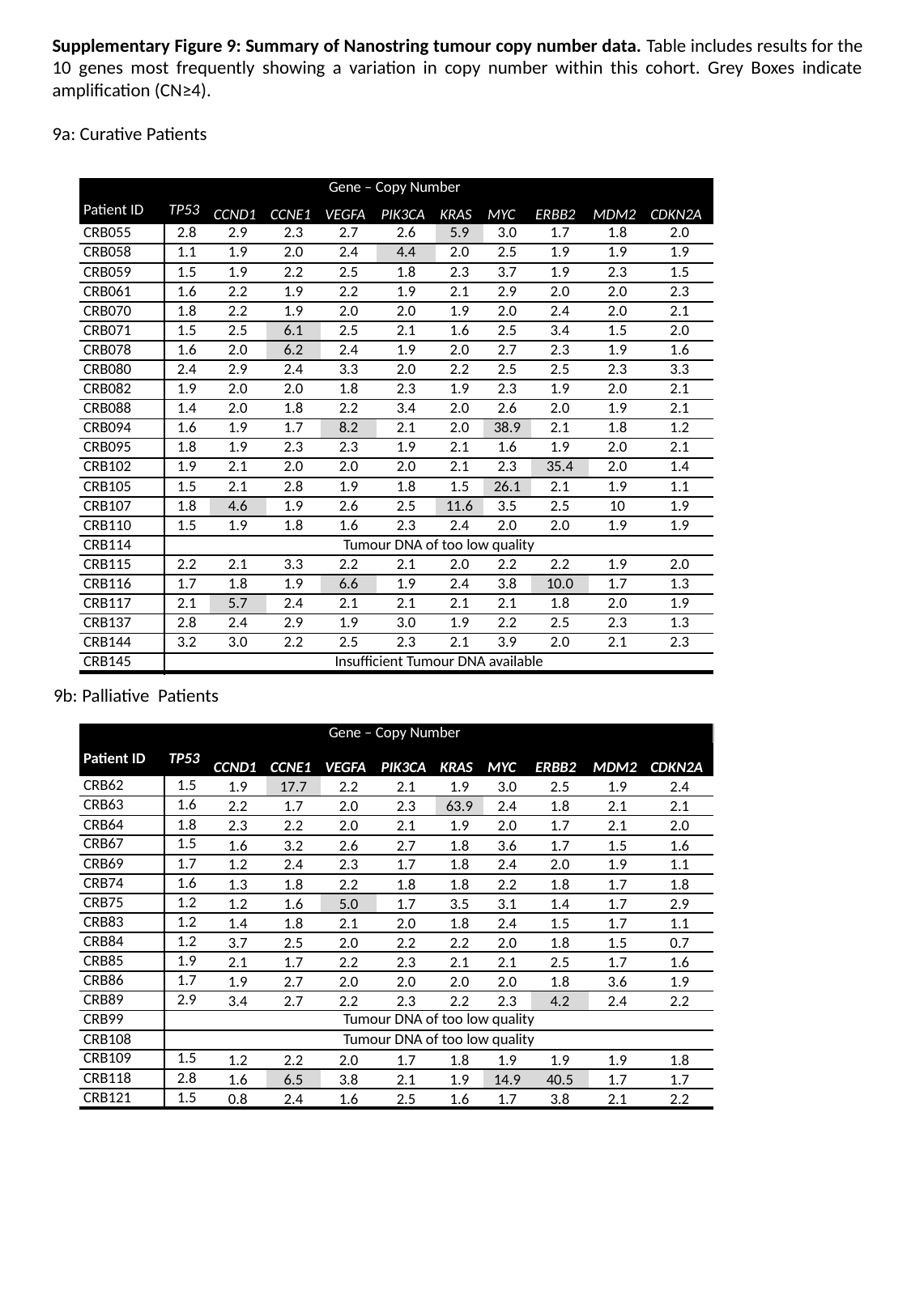

Supplementary Figure 9: Summary of Nanostring tumour copy number data. Table includes results for the 10 genes most frequently showing a variation in copy number within this cohort. Grey Boxes indicate amplification (CN≥4).
9a: Curative Patients
| Gene – Copy Number | | | | | | | | | | |
| --- | --- | --- | --- | --- | --- | --- | --- | --- | --- | --- |
| Patient ID | TP53 | CCND1 | CCNE1 | VEGFA | PIK3CA | KRAS | MYC | ERBB2 | MDM2 | CDKN2A |
| CRB055 | 2.8 | 2.9 | 2.3 | 2.7 | 2.6 | 5.9 | 3.0 | 1.7 | 1.8 | 2.0 |
| CRB058 | 1.1 | 1.9 | 2.0 | 2.4 | 4.4 | 2.0 | 2.5 | 1.9 | 1.9 | 1.9 |
| CRB059 | 1.5 | 1.9 | 2.2 | 2.5 | 1.8 | 2.3 | 3.7 | 1.9 | 2.3 | 1.5 |
| CRB061 | 1.6 | 2.2 | 1.9 | 2.2 | 1.9 | 2.1 | 2.9 | 2.0 | 2.0 | 2.3 |
| CRB070 | 1.8 | 2.2 | 1.9 | 2.0 | 2.0 | 1.9 | 2.0 | 2.4 | 2.0 | 2.1 |
| CRB071 | 1.5 | 2.5 | 6.1 | 2.5 | 2.1 | 1.6 | 2.5 | 3.4 | 1.5 | 2.0 |
| CRB078 | 1.6 | 2.0 | 6.2 | 2.4 | 1.9 | 2.0 | 2.7 | 2.3 | 1.9 | 1.6 |
| CRB080 | 2.4 | 2.9 | 2.4 | 3.3 | 2.0 | 2.2 | 2.5 | 2.5 | 2.3 | 3.3 |
| CRB082 | 1.9 | 2.0 | 2.0 | 1.8 | 2.3 | 1.9 | 2.3 | 1.9 | 2.0 | 2.1 |
| CRB088 | 1.4 | 2.0 | 1.8 | 2.2 | 3.4 | 2.0 | 2.6 | 2.0 | 1.9 | 2.1 |
| CRB094 | 1.6 | 1.9 | 1.7 | 8.2 | 2.1 | 2.0 | 38.9 | 2.1 | 1.8 | 1.2 |
| CRB095 | 1.8 | 1.9 | 2.3 | 2.3 | 1.9 | 2.1 | 1.6 | 1.9 | 2.0 | 2.1 |
| CRB102 | 1.9 | 2.1 | 2.0 | 2.0 | 2.0 | 2.1 | 2.3 | 35.4 | 2.0 | 1.4 |
| CRB105 | 1.5 | 2.1 | 2.8 | 1.9 | 1.8 | 1.5 | 26.1 | 2.1 | 1.9 | 1.1 |
| CRB107 | 1.8 | 4.6 | 1.9 | 2.6 | 2.5 | 11.6 | 3.5 | 2.5 | 10 | 1.9 |
| CRB110 | 1.5 | 1.9 | 1.8 | 1.6 | 2.3 | 2.4 | 2.0 | 2.0 | 1.9 | 1.9 |
| CRB114 | Tumour DNA of too low quality | | | | | | | | | |
| CRB115 | 2.2 | 2.1 | 3.3 | 2.2 | 2.1 | 2.0 | 2.2 | 2.2 | 1.9 | 2.0 |
| CRB116 | 1.7 | 1.8 | 1.9 | 6.6 | 1.9 | 2.4 | 3.8 | 10.0 | 1.7 | 1.3 |
| CRB117 | 2.1 | 5.7 | 2.4 | 2.1 | 2.1 | 2.1 | 2.1 | 1.8 | 2.0 | 1.9 |
| CRB137 | 2.8 | 2.4 | 2.9 | 1.9 | 3.0 | 1.9 | 2.2 | 2.5 | 2.3 | 1.3 |
| CRB144 | 3.2 | 3.0 | 2.2 | 2.5 | 2.3 | 2.1 | 3.9 | 2.0 | 2.1 | 2.3 |
| CRB145 | Insufficient Tumour DNA available | | | | | | | | | |
9b: Palliative Patients
| Gene – Copy Number | | | | | | | | | | |
| --- | --- | --- | --- | --- | --- | --- | --- | --- | --- | --- |
| Patient ID | TP53 | CCND1 | CCNE1 | VEGFA | PIK3CA | KRAS | MYC | ERBB2 | MDM2 | CDKN2A |
| CRB62 | 1.5 | 1.9 | 17.7 | 2.2 | 2.1 | 1.9 | 3.0 | 2.5 | 1.9 | 2.4 |
| CRB63 | 1.6 | 2.2 | 1.7 | 2.0 | 2.3 | 63.9 | 2.4 | 1.8 | 2.1 | 2.1 |
| CRB64 | 1.8 | 2.3 | 2.2 | 2.0 | 2.1 | 1.9 | 2.0 | 1.7 | 2.1 | 2.0 |
| CRB67 | 1.5 | 1.6 | 3.2 | 2.6 | 2.7 | 1.8 | 3.6 | 1.7 | 1.5 | 1.6 |
| CRB69 | 1.7 | 1.2 | 2.4 | 2.3 | 1.7 | 1.8 | 2.4 | 2.0 | 1.9 | 1.1 |
| CRB74 | 1.6 | 1.3 | 1.8 | 2.2 | 1.8 | 1.8 | 2.2 | 1.8 | 1.7 | 1.8 |
| CRB75 | 1.2 | 1.2 | 1.6 | 5.0 | 1.7 | 3.5 | 3.1 | 1.4 | 1.7 | 2.9 |
| CRB83 | 1.2 | 1.4 | 1.8 | 2.1 | 2.0 | 1.8 | 2.4 | 1.5 | 1.7 | 1.1 |
| CRB84 | 1.2 | 3.7 | 2.5 | 2.0 | 2.2 | 2.2 | 2.0 | 1.8 | 1.5 | 0.7 |
| CRB85 | 1.9 | 2.1 | 1.7 | 2.2 | 2.3 | 2.1 | 2.1 | 2.5 | 1.7 | 1.6 |
| CRB86 | 1.7 | 1.9 | 2.7 | 2.0 | 2.0 | 2.0 | 2.0 | 1.8 | 3.6 | 1.9 |
| CRB89 | 2.9 | 3.4 | 2.7 | 2.2 | 2.3 | 2.2 | 2.3 | 4.2 | 2.4 | 2.2 |
| CRB99 | Tumour DNA of too low quality | | | | | | | | | |
| CRB108 | Tumour DNA of too low quality | | | | | | | | | |
| CRB109 | 1.5 | 1.2 | 2.2 | 2.0 | 1.7 | 1.8 | 1.9 | 1.9 | 1.9 | 1.8 |
| CRB118 | 2.8 | 1.6 | 6.5 | 3.8 | 2.1 | 1.9 | 14.9 | 40.5 | 1.7 | 1.7 |
| CRB121 | 1.5 | 0.8 | 2.4 | 1.6 | 2.5 | 1.6 | 1.7 | 3.8 | 2.1 | 2.2 |

## Slide 10
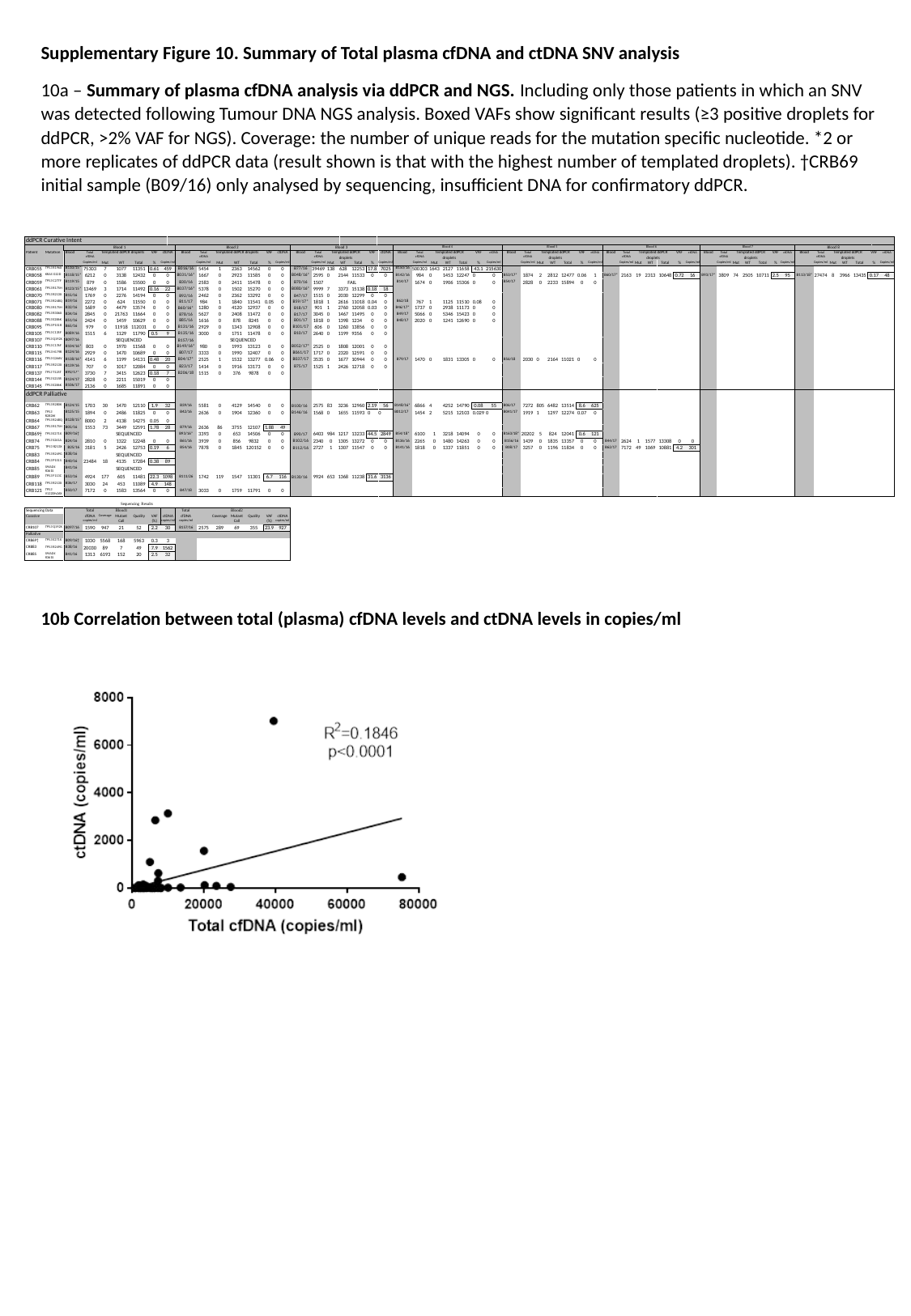

Supplementary Figure 10. Summary of Total plasma cfDNA and ctDNA SNV analysis
10a – Summary of plasma cfDNA analysis via ddPCR and NGS. Including only those patients in which an SNV was detected following Tumour DNA NGS analysis. Boxed VAFs show significant results (≥3 positive droplets for ddPCR, >2% VAF for NGS). Coverage: the number of unique reads for the mutation specific nucleotide. *2 or more replicates of ddPCR data (result shown is that with the highest number of templated droplets). †CRB69 initial sample (B09/16) only analysed by sequencing, insufficient DNA for confirmatory ddPCR.
10b Correlation between total (plasma) cfDNA levels and ctDNA levels in copies/ml

## Slide 11
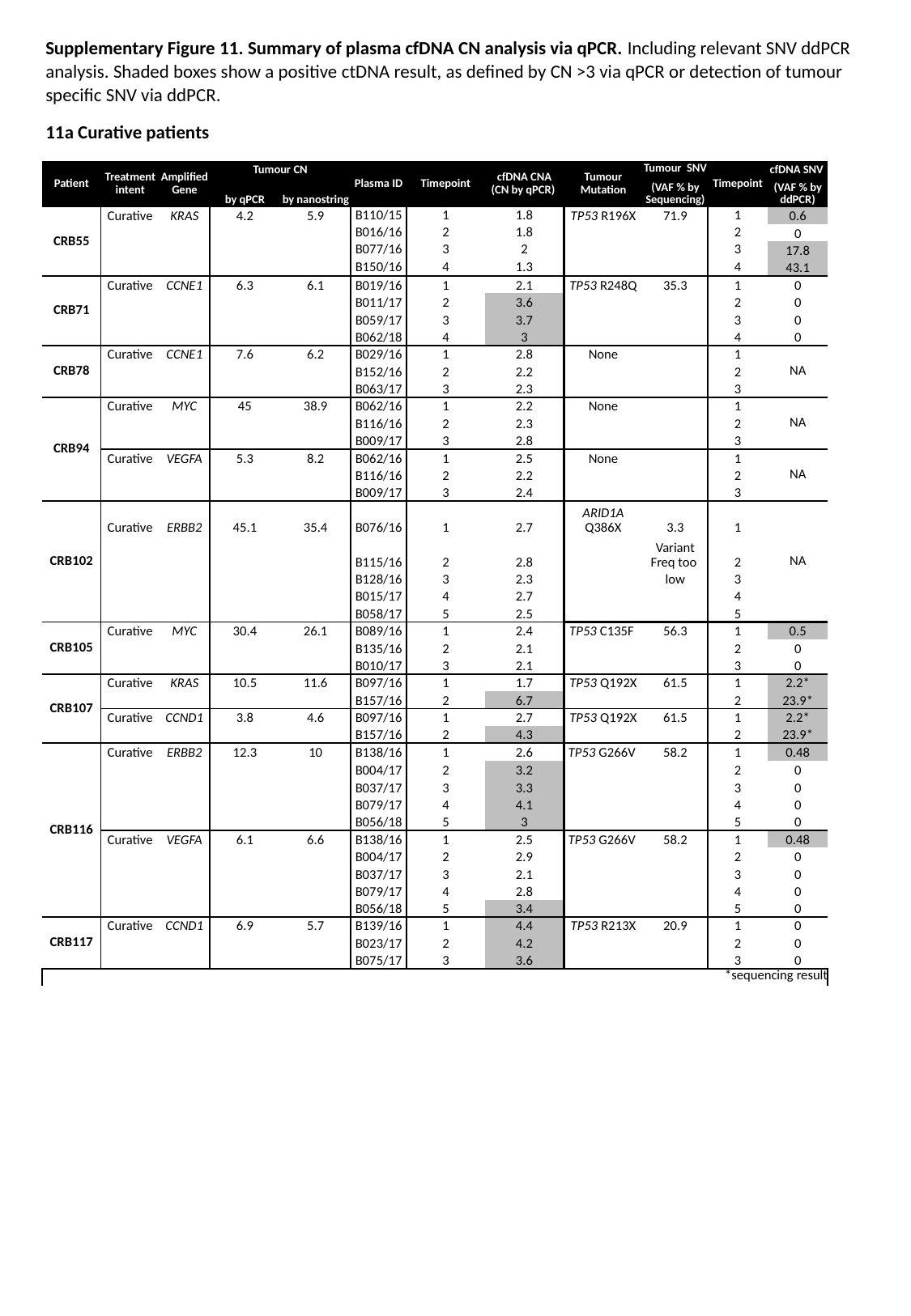

Supplementary Figure 11. Summary of plasma cfDNA CN analysis via qPCR. Including relevant SNV ddPCR analysis. Shaded boxes show a positive ctDNA result, as defined by CN >3 via qPCR or detection of tumour specific SNV via ddPCR.
11a Curative patients
| Patient | Treatment intent | Amplified Gene | Tumour CN | | Plasma ID | Timepoint | cfDNA CNA (CN by qPCR) | Tumour Mutation | Tumour SNV | Timepoint | cfDNA SNV |
| --- | --- | --- | --- | --- | --- | --- | --- | --- | --- | --- | --- |
| | | | by qPCR | by nanostring | | | | | (VAF % by Sequencing) | | (VAF % by ddPCR) |
| CRB55 | Curative | KRAS | 4.2 | 5.9 | B110/15 | 1 | 1.8 | TP53 R196X | 71.9 | 1 | 0.6 |
| | | | | | B016/16 | 2 | 1.8 | | | 2 | 0 |
| | | | | | B077/16 | 3 | 2 | | | 3 | 17.8 |
| | | | | | B150/16 | 4 | 1.3 | | | 4 | 43.1 |
| CRB71 | Curative | CCNE1 | 6.3 | 6.1 | B019/16 | 1 | 2.1 | TP53 R248Q | 35.3 | 1 | 0 |
| | | | | | B011/17 | 2 | 3.6 | | | 2 | 0 |
| | | | | | B059/17 | 3 | 3.7 | | | 3 | 0 |
| | | | | | B062/18 | 4 | 3 | | | 4 | 0 |
| CRB78 | Curative | CCNE1 | 7.6 | 6.2 | B029/16 | 1 | 2.8 | None | | 1 | NA |
| | | | | | B152/16 | 2 | 2.2 | | | 2 | |
| | | | | | B063/17 | 3 | 2.3 | | | 3 | |
| CRB94 | Curative | MYC | 45 | 38.9 | B062/16 | 1 | 2.2 | None | | 1 | NA |
| | | | | | B116/16 | 2 | 2.3 | | | 2 | |
| | | | | | B009/17 | 3 | 2.8 | | | 3 | |
| | Curative | VEGFA | 5.3 | 8.2 | B062/16 | 1 | 2.5 | None | | 1 | NA |
| | | | | | B116/16 | 2 | 2.2 | | | 2 | |
| | | | | | B009/17 | 3 | 2.4 | | | 3 | |
| CRB102 | Curative | ERBB2 | 45.1 | 35.4 | B076/16 | 1 | 2.7 | ARID1A Q386X | 3.3 | 1 | NA |
| | | | | | B115/16 | 2 | 2.8 | | Variant Freq too | 2 | |
| | | | | | B128/16 | 3 | 2.3 | | low | 3 | |
| | | | | | B015/17 | 4 | 2.7 | | | 4 | |
| | | | | | B058/17 | 5 | 2.5 | | | 5 | |
| CRB105 | Curative | MYC | 30.4 | 26.1 | B089/16 | 1 | 2.4 | TP53 C135F | 56.3 | 1 | 0.5 |
| | | | | | B135/16 | 2 | 2.1 | | | 2 | 0 |
| | | | | | B010/17 | 3 | 2.1 | | | 3 | 0 |
| CRB107 | Curative | KRAS | 10.5 | 11.6 | B097/16 | 1 | 1.7 | TP53 Q192X | 61.5 | 1 | 2.2\* |
| | | | | | B157/16 | 2 | 6.7 | | | 2 | 23.9\* |
| | Curative | CCND1 | 3.8 | 4.6 | B097/16 | 1 | 2.7 | TP53 Q192X | 61.5 | 1 | 2.2\* |
| | | | | | B157/16 | 2 | 4.3 | | | 2 | 23.9\* |
| CRB116 | Curative | ERBB2 | 12.3 | 10 | B138/16 | 1 | 2.6 | TP53 G266V | 58.2 | 1 | 0.48 |
| | | | | | B004/17 | 2 | 3.2 | | | 2 | 0 |
| | | | | | B037/17 | 3 | 3.3 | | | 3 | 0 |
| | | | | | B079/17 | 4 | 4.1 | | | 4 | 0 |
| | | | | | B056/18 | 5 | 3 | | | 5 | 0 |
| | Curative | VEGFA | 6.1 | 6.6 | B138/16 | 1 | 2.5 | TP53 G266V | 58.2 | 1 | 0.48 |
| | | | | | B004/17 | 2 | 2.9 | | | 2 | 0 |
| | | | | | B037/17 | 3 | 2.1 | | | 3 | 0 |
| | | | | | B079/17 | 4 | 2.8 | | | 4 | 0 |
| | | | | | B056/18 | 5 | 3.4 | | | 5 | 0 |
| CRB117 | Curative | CCND1 | 6.9 | 5.7 | B139/16 | 1 | 4.4 | TP53 R213X | 20.9 | 1 | 0 |
| | | | | | B023/17 | 2 | 4.2 | | | 2 | 0 |
| | | | | | B075/17 | 3 | 3.6 | | | 3 | 0 |
| \*sequencing result | | | | | | | | | | | |

## Slide 12
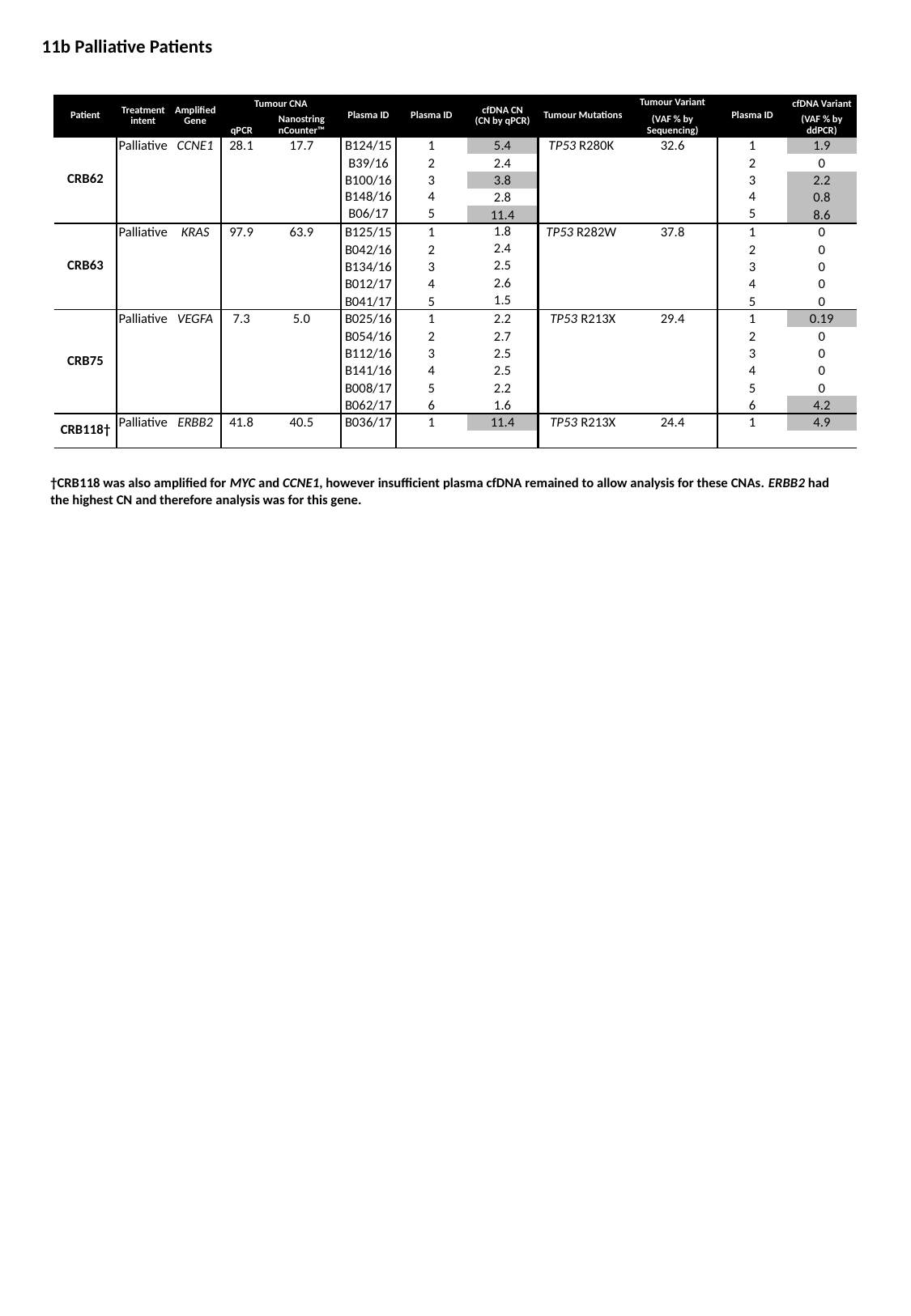

11b Palliative Patients
| Patient | Treatment intent | Amplified Gene | Tumour CNA | | Plasma ID | Plasma ID | cfDNA CN (CN by qPCR) | Tumour Mutations | Tumour Variant | Plasma ID | cfDNA Variant |
| --- | --- | --- | --- | --- | --- | --- | --- | --- | --- | --- | --- |
| | | | qPCR | Nanostring nCounter™ | | | | | (VAF % by Sequencing) | | (VAF % by ddPCR) |
| CRB62 | Palliative | CCNE1 | 28.1 | 17.7 | B124/15 | 1 | 5.4 | TP53 R280K | 32.6 | 1 | 1.9 |
| | | | | | B39/16 | 2 | 2.4 | | | 2 | 0 |
| | | | | | B100/16 | 3 | 3.8 | | | 3 | 2.2 |
| | | | | | B148/16 | 4 | 2.8 | | | 4 | 0.8 |
| | | | | | B06/17 | 5 | 11.4 | | | 5 | 8.6 |
| CRB63 | Palliative | KRAS | 97.9 | 63.9 | B125/15 | 1 | 1.8 | TP53 R282W | 37.8 | 1 | 0 |
| | | | | | B042/16 | 2 | 2.4 | | | 2 | 0 |
| | | | | | B134/16 | 3 | 2.5 | | | 3 | 0 |
| | | | | | B012/17 | 4 | 2.6 | | | 4 | 0 |
| | | | | | B041/17 | 5 | 1.5 | | | 5 | 0 |
| CRB75 | Palliative | VEGFA | 7.3 | 5.0 | B025/16 | 1 | 2.2 | TP53 R213X | 29.4 | 1 | 0.19 |
| | | | | | B054/16 | 2 | 2.7 | | | 2 | 0 |
| | | | | | B112/16 | 3 | 2.5 | | | 3 | 0 |
| | | | | | B141/16 | 4 | 2.5 | | | 4 | 0 |
| | | | | | B008/17 | 5 | 2.2 | | | 5 | 0 |
| | | | | | B062/17 | 6 | 1.6 | | | 6 | 4.2 |
| CRB118† | Palliative | ERBB2 | 41.8 | 40.5 | B036/17 | 1 | 11.4 | TP53 R213X | 24.4 | 1 | 4.9 |
| | | | | | | | | | | | |
†CRB118 was also amplified for MYC and CCNE1, however insufficient plasma cfDNA remained to allow analysis for these CNAs. ERBB2 had the highest CN and therefore analysis was for this gene.

## Slide 13
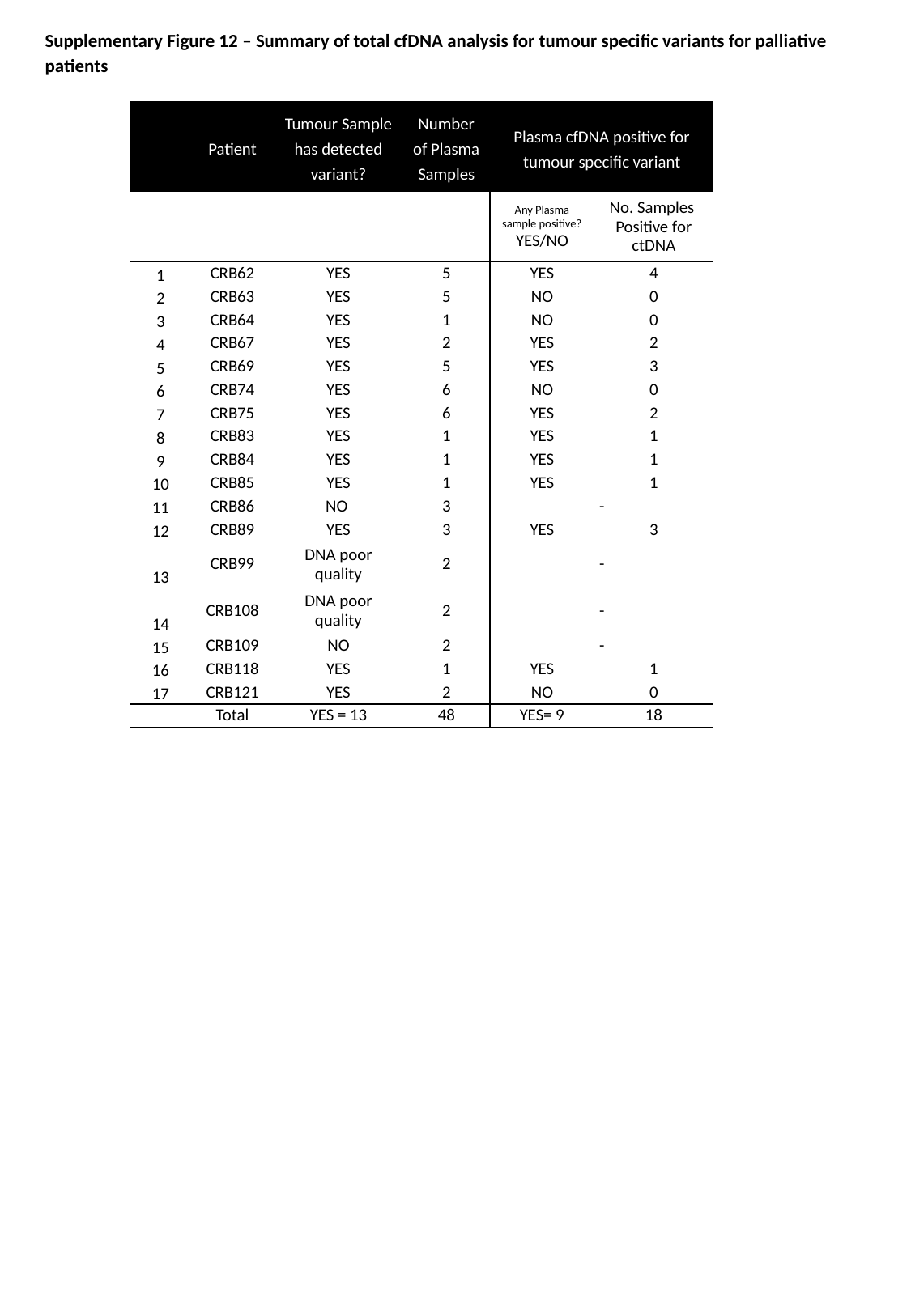

Supplementary Figure 12 – Summary of total cfDNA analysis for tumour specific variants for palliative patients
| | Patient | Tumour Sample has detected variant? | Number of Plasma Samples | Plasma cfDNA positive for tumour specific variant | |
| --- | --- | --- | --- | --- | --- |
| | | | | Any Plasma sample positive? YES/NO | No. Samples Positive for ctDNA |
| 1 | CRB62 | YES | 5 | YES | 4 |
| 2 | CRB63 | YES | 5 | NO | 0 |
| 3 | CRB64 | YES | 1 | NO | 0 |
| 4 | CRB67 | YES | 2 | YES | 2 |
| 5 | CRB69 | YES | 5 | YES | 3 |
| 6 | CRB74 | YES | 6 | NO | 0 |
| 7 | CRB75 | YES | 6 | YES | 2 |
| 8 | CRB83 | YES | 1 | YES | 1 |
| 9 | CRB84 | YES | 1 | YES | 1 |
| 10 | CRB85 | YES | 1 | YES | 1 |
| 11 | CRB86 | NO | 3 | - | |
| 12 | CRB89 | YES | 3 | YES | 3 |
| 13 | CRB99 | DNA poor quality | 2 | - | |
| 14 | CRB108 | DNA poor quality | 2 | - | |
| 15 | CRB109 | NO | 2 | - | |
| 16 | CRB118 | YES | 1 | YES | 1 |
| 17 | CRB121 | YES | 2 | NO | 0 |
| | Total | YES = 13 | 48 | YES= 9 | 18 |

## Slide 14
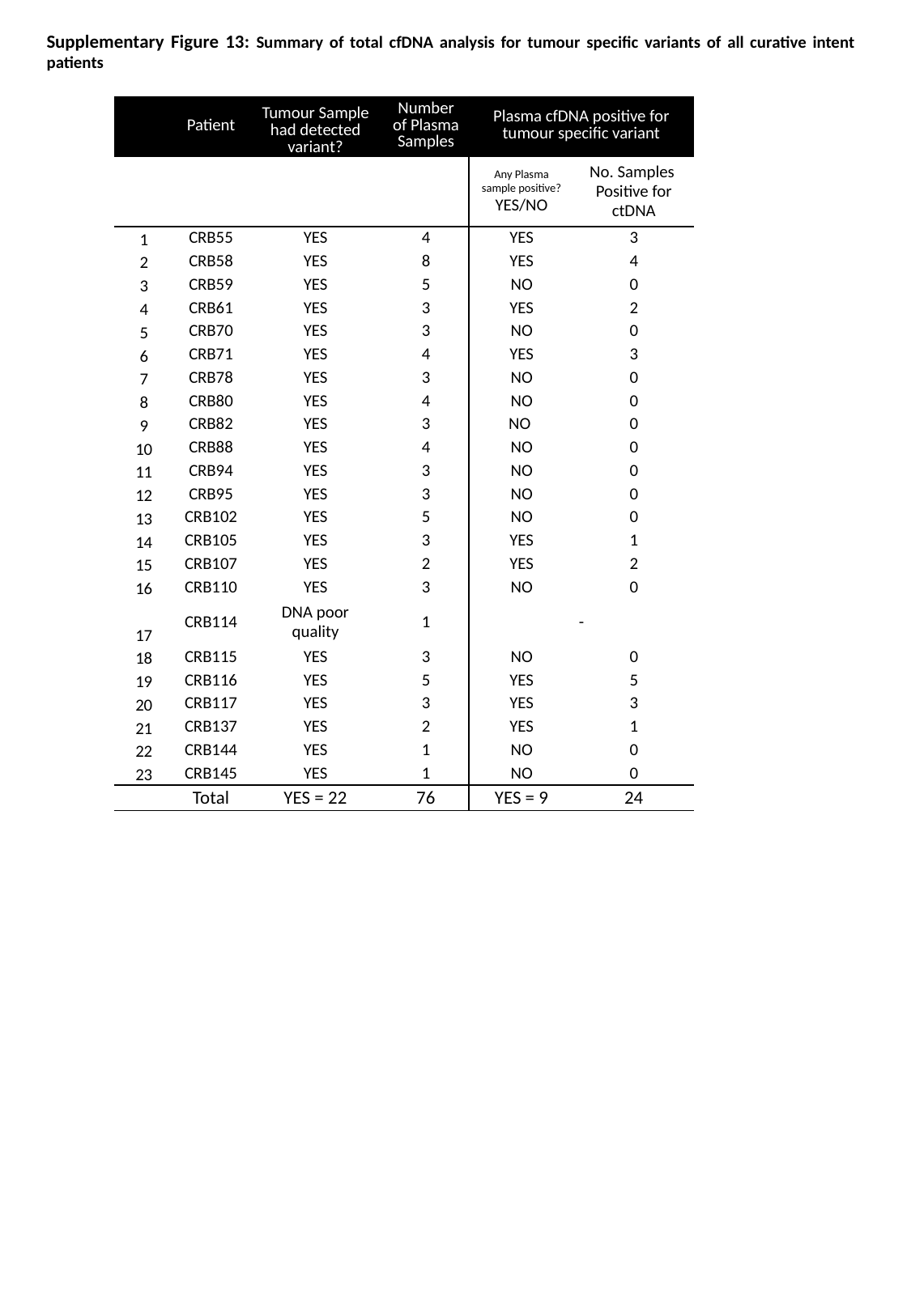

Supplementary Figure 13: Summary of total cfDNA analysis for tumour specific variants of all curative intent patients
| | Patient | Tumour Sample had detected variant? | Number of Plasma Samples | Plasma cfDNA positive for tumour specific variant | |
| --- | --- | --- | --- | --- | --- |
| | | | | Any Plasma sample positive? YES/NO | No. Samples Positive for ctDNA |
| 1 | CRB55 | YES | 4 | YES | 3 |
| 2 | CRB58 | YES | 8 | YES | 4 |
| 3 | CRB59 | YES | 5 | NO | 0 |
| 4 | CRB61 | YES | 3 | YES | 2 |
| 5 | CRB70 | YES | 3 | NO | 0 |
| 6 | CRB71 | YES | 4 | YES | 3 |
| 7 | CRB78 | YES | 3 | NO | 0 |
| 8 | CRB80 | YES | 4 | NO | 0 |
| 9 | CRB82 | YES | 3 | NO | 0 |
| 10 | CRB88 | YES | 4 | NO | 0 |
| 11 | CRB94 | YES | 3 | NO | 0 |
| 12 | CRB95 | YES | 3 | NO | 0 |
| 13 | CRB102 | YES | 5 | NO | 0 |
| 14 | CRB105 | YES | 3 | YES | 1 |
| 15 | CRB107 | YES | 2 | YES | 2 |
| 16 | CRB110 | YES | 3 | NO | 0 |
| 17 | CRB114 | DNA poor quality | 1 | - | |
| 18 | CRB115 | YES | 3 | NO | 0 |
| 19 | CRB116 | YES | 5 | YES | 5 |
| 20 | CRB117 | YES | 3 | YES | 3 |
| 21 | CRB137 | YES | 2 | YES | 1 |
| 22 | CRB144 | YES | 1 | NO | 0 |
| 23 | CRB145 | YES | 1 | NO | 0 |
| | Total | YES = 22 | 76 | YES = 9 | 24 |

## Slide 15
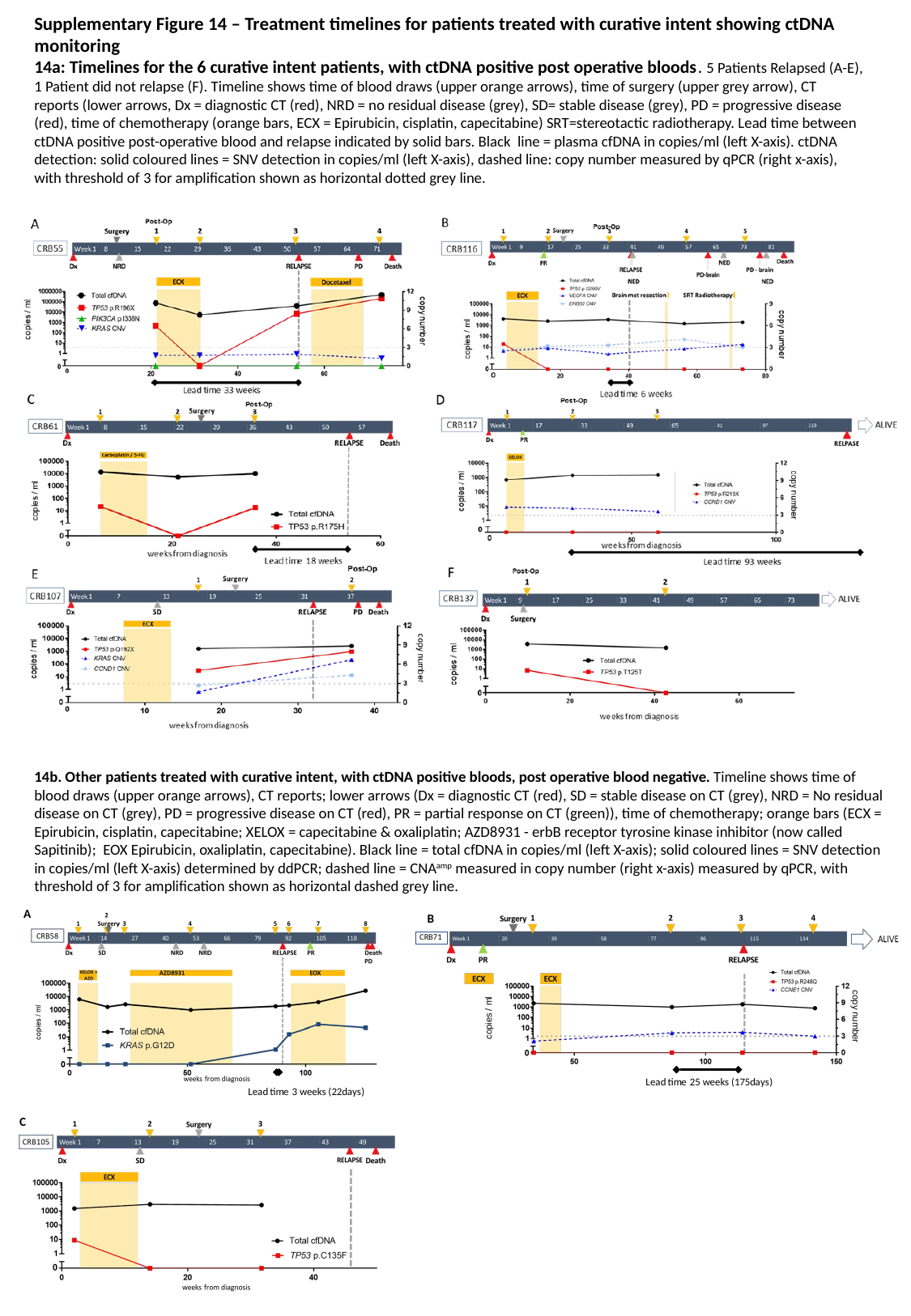

Supplementary Figure 14 – Treatment timelines for patients treated with curative intent showing ctDNA monitoring
14a: Timelines for the 6 curative intent patients, with ctDNA positive post operative bloods. 5 Patients Relapsed (A-E), 1 Patient did not relapse (F). Timeline shows time of blood draws (upper orange arrows), time of surgery (upper grey arrow), CT reports (lower arrows, Dx = diagnostic CT (red), NRD = no residual disease (grey), SD= stable disease (grey), PD = progressive disease (red), time of chemotherapy (orange bars, ECX = Epirubicin, cisplatin, capecitabine) SRT=stereotactic radiotherapy. Lead time between ctDNA positive post-operative blood and relapse indicated by solid bars. Black line = plasma cfDNA in copies/ml (left X-axis). ctDNA detection: solid coloured lines = SNV detection in copies/ml (left X-axis), dashed line: copy number measured by qPCR (right x-axis), with threshold of 3 for amplification shown as horizontal dotted grey line.
14b. Other patients treated with curative intent, with ctDNA positive bloods, post operative blood negative. Timeline shows time of blood draws (upper orange arrows), CT reports; lower arrows (Dx = diagnostic CT (red), SD = stable disease on CT (grey), NRD = No residual disease on CT (grey), PD = progressive disease on CT (red), PR = partial response on CT (green)), time of chemotherapy; orange bars (ECX = Epirubicin, cisplatin, capecitabine; XELOX = capecitabine & oxaliplatin; AZD8931 - erbB receptor tyrosine kinase inhibitor (now called Sapitinib); EOX Epirubicin, oxaliplatin, capecitabine). Black line = total cfDNA in copies/ml (left X-axis); solid coloured lines = SNV detection in copies/ml (left X-axis) determined by ddPCR; dashed line = CNAamp measured in copy number (right x-axis) measured by qPCR, with threshold of 3 for amplification shown as horizontal dashed grey line.

## Slide 16
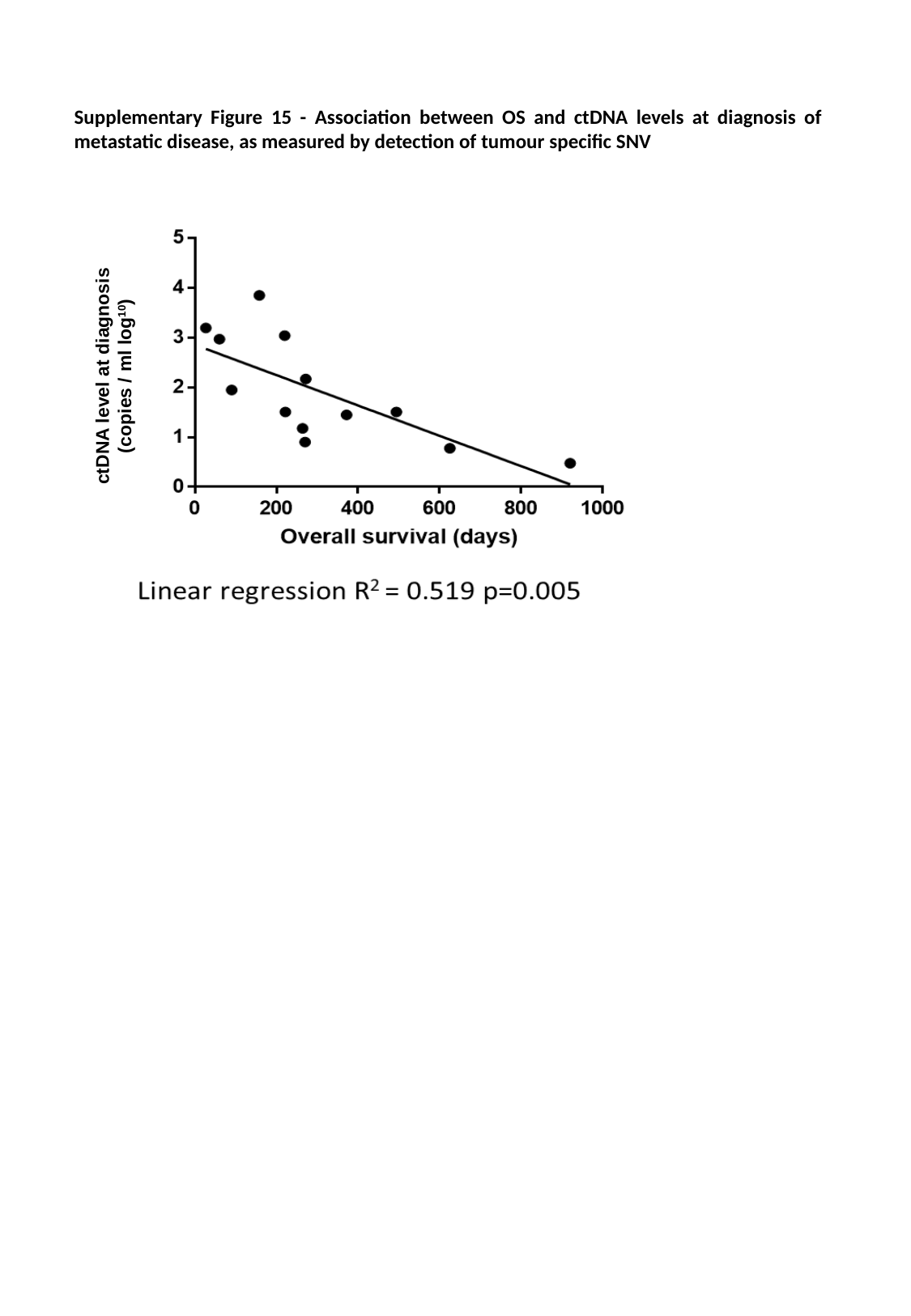

Supplementary Figure 15 - Association between OS and ctDNA levels at diagnosis of metastatic disease, as measured by detection of tumour specific SNV
ctDNA level at diagnosis
(copies / ml log10)

## Slide 17
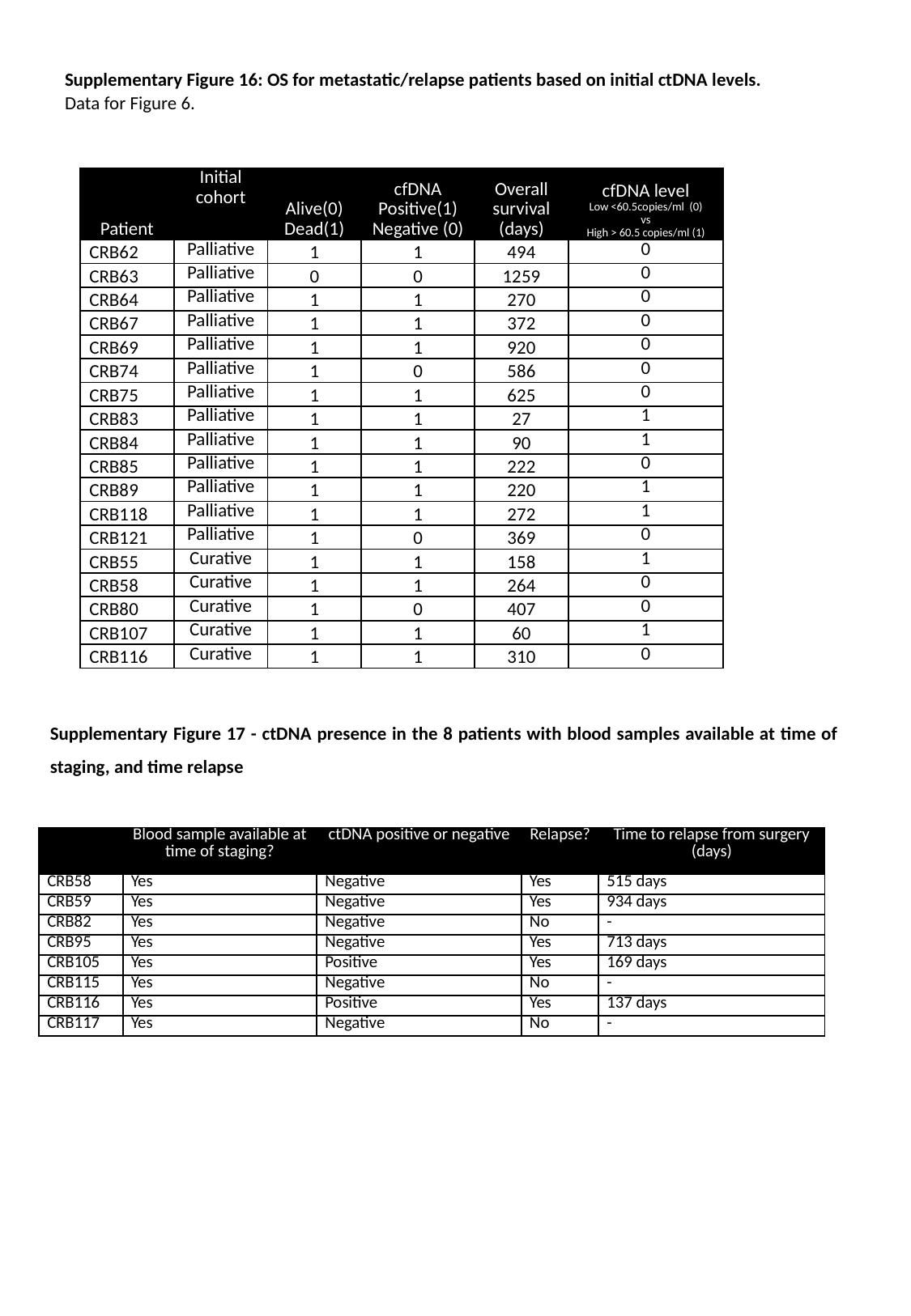

Supplementary Figure 16: OS for metastatic/relapse patients based on initial ctDNA levels. Data for Figure 6.
| Patient | Initial cohort | Alive(0) Dead(1) | cfDNA Positive(1) Negative (0) | Overall survival (days) | cfDNA level Low <60.5copies/ml (0) vs High > 60.5 copies/ml (1) |
| --- | --- | --- | --- | --- | --- |
| CRB62 | Palliative | 1 | 1 | 494 | 0 |
| CRB63 | Palliative | 0 | 0 | 1259 | 0 |
| CRB64 | Palliative | 1 | 1 | 270 | 0 |
| CRB67 | Palliative | 1 | 1 | 372 | 0 |
| CRB69 | Palliative | 1 | 1 | 920 | 0 |
| CRB74 | Palliative | 1 | 0 | 586 | 0 |
| CRB75 | Palliative | 1 | 1 | 625 | 0 |
| CRB83 | Palliative | 1 | 1 | 27 | 1 |
| CRB84 | Palliative | 1 | 1 | 90 | 1 |
| CRB85 | Palliative | 1 | 1 | 222 | 0 |
| CRB89 | Palliative | 1 | 1 | 220 | 1 |
| CRB118 | Palliative | 1 | 1 | 272 | 1 |
| CRB121 | Palliative | 1 | 0 | 369 | 0 |
| CRB55 | Curative | 1 | 1 | 158 | 1 |
| CRB58 | Curative | 1 | 1 | 264 | 0 |
| CRB80 | Curative | 1 | 0 | 407 | 0 |
| CRB107 | Curative | 1 | 1 | 60 | 1 |
| CRB116 | Curative | 1 | 1 | 310 | 0 |
Supplementary Figure 17 - ctDNA presence in the 8 patients with blood samples available at time of staging, and time relapse
| Patient | Blood sample available at time of staging? | ctDNA positive or negative | Relapse? | Time to relapse from surgery (days) |
| --- | --- | --- | --- | --- |
| CRB58 | Yes | Negative | Yes | 515 days |
| CRB59 | Yes | Negative | Yes | 934 days |
| CRB82 | Yes | Negative | No | - |
| CRB95 | Yes | Negative | Yes | 713 days |
| CRB105 | Yes | Positive | Yes | 169 days |
| CRB115 | Yes | Negative | No | - |
| CRB116 | Yes | Positive | Yes | 137 days |
| CRB117 | Yes | Negative | No | - |
